# The host transcriptional response to Candidemia is dominated by neutrophil activation and heme biosynthesis and supports novel diagnostic approaches

**DOI:** 10.1186/s13073-021-00924-9

**Published:** 2021-07-05

**Authors:** Julie M. Steinbrink, Rachel A. Myers, Kaiyuan Hua, Melissa D. Johnson, Jessica L. Seidelman, Ephraim L. Tsalik, Ricardo Henao, Geoffrey S. Ginsburg, Christopher W. Woods, Barbara D. Alexander, Micah T. McClain

**Affiliations:** 1 Division of Infectious Diseases, Duke University Medical Center, Durham, NC USA; 2 Center for Applied Genomics and Precision Medicine, Duke University, Durham, NC USA; 3 Emergency Medicine Service, Durham Veterans Affairs Health Care System, Durham, NC USA; 4 Division of Infectious Diseases, Durham Veterans Affairs Health Care System, Durham, NC USA

**Keywords:** Candidemia, Gene expression, Biomarkers, Host response, Fungal diagnostics

## Abstract

**Background:**

Candidemia is one of the most common nosocomial bloodstream infections in the United States, causing significant morbidity and mortality in hospitalized patients, but the breadth of the host response to *Candida* infections in human patients remains poorly defined.

**Methods:**

In order to better define the host response to *Candida* infection at the transcriptional level, we performed RNA sequencing on serial peripheral blood samples from 48 hospitalized patients with blood cultures positive for *Candida* species and compared them to patients with other acute viral, bacterial, and non-infectious illnesses. Regularized multinomial regression was utilized to develop pathogen class-specific gene expression classifiers.

**Results:**

Candidemia triggers a unique, robust, and conserved transcriptomic response in human hosts with 1641 genes differentially upregulated compared to healthy controls. Many of these genes corresponded to components of the immune response to fungal infection, heavily weighted toward neutrophil activation, heme biosynthesis, and T cell signaling. We developed pathogen class-specific classifiers from these unique signals capable of identifying and differentiating candidemia, viral, or bacterial infection across a variety of hosts with a high degree of accuracy (auROC 0.98 for candidemia, 0.99 for viral and bacterial infection). This classifier was validated on two separate human cohorts (auROC 0.88 for viral infection and 0.87 for bacterial infection in one cohort; auROC 0.97 in another cohort) and an in vitro model (auROC 0.94 for fungal infection, 0.96 for bacterial, and 0.90 for viral infection).

**Conclusions:**

Transcriptional analysis of circulating leukocytes in patients with acute *Candida* infections defines novel aspects of the breadth of the human immune response during candidemia and suggests promising diagnostic approaches for simultaneously differentiating multiple types of clinical illnesses in at-risk, acutely ill patients.

**Supplementary Information:**

The online version contains supplementary material available at 10.1186/s13073-021-00924-9.

## Background

Candidemia is one of the most common nosocomial bloodstream infections in the United States and its prevalence continues to increase [1–3]. It has been widely shown to cause significant morbidity and mortality in hospitalized patients [4–9]. Bloodstream infection with *Candida* occurs more commonly in critically ill patients in intensive care units (ICUs) often with multiple underlying medical comorbidities.

Unfortunately, it is difficult to differentiate candidemia from other infections at the time of disease onset, which delays patients’ access to appropriate antimicrobial therapy [10]. The gold standard diagnostic test for candidemia is the blood culture. However, blood cultures suffer from variable sensitivity and a delay to positivity [11–13]. This has led to the development of additional laboratory markers of fungal infection, including serum 1,3-beta-d-glucan (BDG)—a cell wall component of many yeasts and molds. However, the sensitivity and specificity of this test varies widely based on clinical circumstances [14, 15]. Newer technologies based on direct molecular detection of pathogens in clinical specimens such as the T2 Candida^TM^ panel and metagenomic approaches from Karius^TM^ and IDbyDNA^TM^ are promising but also have limitations, including expense and the potential for false-positive results [16–18]. Due to the inadequacies in currently available methods, improved diagnostic approaches are clearly needed.

One such approach is the utilization of host-based gene expression profiles, which can provide pathogen-agnostic information about multiple types of infection [19]. Furthermore, when migrated to a polymerase chain reaction (PCR)-based platforms that are routinely available in clinical microbiology labs, these techniques offer the potential for providing rapid, even point-of-care diagnostic information [20, 21]. This capability has been extensively demonstrated with viral and bacterial causes of respiratory infection. However, little is known about how this approach performs in the setting of fungal disease [19, 20, 22–26]. When available, such a rapid test could decrease the time to more targeted therapy, which positively impacts patient outcomes including length of hospitalization and mortality [27–29]. It could also promote improved antimicrobial stewardship by reducing the amount of time a patient is exposed to inappropriate antimicrobials [30, 31].

To define the utility of host-based biomarkers for diagnosis of candidemia in human subjects and the ability of such a classifier to discriminate between fungal infection and other pathogen classes, we examined transcriptomic responses in a cohort of patients with culture-confirmed *Candida* blood infection compared with other acute infectious and non-infectious illnesses. A transcriptomic signature specific for each pathogen class was generated.

## Methods

### Subject enrollment

All study patients were enrolled after written informed consent at the Duke University Medical Center (DUMC). The study was approved by the Institutional Review Board (IRB) at DUMC (Pro00083484) and was performed in accordance with the Declaration of Helsinki. Forty-eight hospitalized patients with candidemia were enrolled through the Infectious Diseases Data and Specimen Repository program at Duke University (Durham, NC) at the time of first blood culture positivity for *Candida* spp. between the years 2011 and 2014. Whole blood was collected from these subjects in PAXGene tubes for RNA sequencing and serum was collected from each subject for additional analysis. Samples were collected approximately every 2–3 days until blood cultures cleared. Each subject with candidemia had at least 1 and at most 14 samples collected over the course of the study. RNA sequencing data from previously enrolled subjects presenting to the Emergency Department with viral, bacterial, or non-infectious illness (from DUMC, Durham VA Health Care System, UNC Health Care, and Henry Ford Hospital) were also run with the candidemia samples, at a single timepoint per subject [19]. Peripheral blood samples were also similarly collected at a single timepoint per subject from a population of 30 non-hospitalized healthy controls enrolled at Duke University.

All subjects were adjudicated for etiology of acute illness by a panel of infectious diseases specialists by retrospective manual chart review, after enrollment but prior to gene expression measurements. Phenotype classification was made if a subject had both the signs/symptoms of an infectious disease and an identified pathogen compatible with their clinical syndrome based on available clinical, laboratory, and microbiologic data. The adjudication process used here has been previously described in detail [19, 32]. Non-infectious subjects were labeled as a systemic inflammatory response syndrome (SIRS) phenotype—defined by at least two SIRS criteria (temperature <36°C (C) or >38°C, tachycardia >90 beats per minute, tachypnea >20 breaths per minute or PaCO_2_ <32 mmHg, white cell count <4000 cells/mm^3^, or >12,000 cells/mm^3^ or >10% neutrophil band forms) without evidence of infection.

**Fig. 1:**
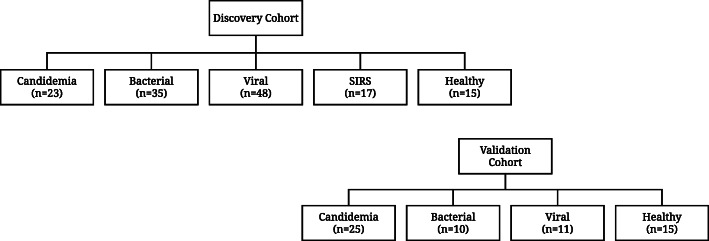
Experimental design. Breakdown of discovery and validation cohorts by infection phenotype

Subjects and controls were divided at random into discovery and validation cohorts for initial analysis. The discovery cohort consisted of 138 subjects—23 with bloodstream infection with *Candida* spp. in the absence of other types of infection, 35 with bacterial infection, 48 with a viral infection, 17 with SIRS, and 15 with healthy controls. The validation cohort consisted of 61 subjects—25 with confirmed candidemia, 10 with bacterial infection, 11 with a viral infection, and 15 healthy controls (Fig. 1).

### RNA extraction, library preparation, and sequencing

Total RNA was extracted from the human blood preserved and stored in PAXgene Blood RNA Tubes using the Qiagen PAXgene Blood miRNA Kit according to the manufacturer’s protocol. RNA quantity and quality were assessed using the Nanodrop 2000 spectrophotometer (Thermo Scientific) and Agilent 2100 Bioanalyzer, respectively. RNA sequencing libraries were generated using NuGEN Universal mRNA-seq kit with AnyDeplete Globin (NuGEN Technologies, Redwood City, CA) and sequenced on the Illumina NovaSeq 6000 instrument with S2 flow cell and 50bp paired-end reads (performed through the Duke Sequencing and Genomic Technologies Core).

### RNA sequencing data processing

For both the discovery and validation datasets [33], RNA sequences were mapped to the human genome (hg) and gene expression quantified using STAR with parameters: quantMode: ‘GeneCounts’; outSAMtype: ‘None’; outSAMmode: ‘None’; readFilesCommand: ‘zcat’ and Ensembl gene reference *Homo sapiens* GRCh38 DNA, release 96, downloaded from: ftp://ftp.ensembl.org/pub/release-96/fasta/homo_sapiens/dna/ (for gene quantification) [34]. All other parameters were left at their default values for STAR version 2.7.1a. Samples with a low number of mapped reads (< 12 million reads) or low average pairwise correlation (< 0.70) were excluded from analyses. In the discovery cohort, genes with 0 counts or counts/million < 2 in ≥ 50% of samples were excluded. The validation cohort was reduced to the set of genes passing quality control in the discovery cohort. The remaining gene counts were normalized using TMM, within each cohort.

### Statistical analysis

#### Comparison of clinical demographics

Comparison of clinical demographics was performed by chi-square test for categorical variables or Mann-Whitney for continuous variables.

#### Differential expression

For both the discovery and validation datasets, the R Bioconductor package limma [35] was used to estimate the mean expression for each outcome group: candidemia, bacterial, viral, SIRS, and healthy, while adjusting for age, sex, and race, using the empirical Bayesian linear modeling with voom weights [36]. Generalized linear hypothesis testing (i.e., contrasts) was used to test for differential expression between specific infection-type groups (i.e., candidemia vs. healthy). A false discovery rate of less than 5% was used to determine statistical significance for each comparison. The differential expression results from the discovery and validation cohorts were pooled using inverse-variance weighted combination-analysis of the log_2_ fold changes with a cohort random effect, as implemented in the R package meta.

#### Diagnostic classifier development and validation

Regularized multinomial logistic regression (lasso) [37], implemented in the R package glmnet [38], was used to identify a multi-gene signature of the infection type. We used three different unbiased feature selections prior to constructing the model: (1) top 1000 most variable genes, (2) top 2000 most variable genes, and (3) all ~ 11,100 genes that passed the quality control. The multinomial model performance was estimated using nested leave one sample out cross-validation (LOOCV) as follows: for each sample, one sample was held out and the remaining samples were used to estimate the model. Within the (N-1) samples, 10-fold cross-validation was used to optimize the sparsity parameter. The optimal sparsity parameter was then used to estimate the model in the N-1 samples. (Additional file 1: Supplementary Methods) The resulting model was used to estimate the predicted class probabilities in the held-out samples. After completing the LOOCV, the predicted class probabilities from the held-out samples were used to assess the training performance metrics: per-class auROC, confusion matrices, overall sensitivity, and overall specificity. The overall model was estimated using all data with the sparsity parameter optimized through 10-fold cross-validation of the discovery dataset. This overall model was used to predict infection class probabilities in other sequenced samples from other datasets. Model testing performance metrics included per-class area under the receiver operating characteristics curves (auROCs) and confusion matrices.

#### Additional validation

Independent, external validation was performed with two human microarray gene expression datasets (Tsalik, et al. and Ramilo, et al) [19, 23]. For the Ramilo dataset, Affymetrix CEL files and sample characteristics were downloaded from GEO (GSE6269-GPL96) [39]. CEL files were imported and processed using the R Bioconductor packages readAffy. Expression values were normalized using gcrma. Probes detected in fewer than four samples and Affymetrix control probes were excluded. For the Tsalik dataset, Affymetrix microarray gene expression was previously processed and normalized, as previously described [19, 40]. For both the Ramilo and Tsalik datasets, microarray probes were mapped to Ensembl gene identifiers and reduced to the subset of probes that mapped to the classifier gene list. The resulting expression values were log_2_ transformed and analyzed using the same regularized multinomial modeling, cross-validation procedure, and performance metrics used in the discovery analysis to re-estimate the model weights.

Additional validation was performed with an in vitro PBMC microarray dataset consisting of viral (influenza), bacterial (*Escherichia coli* and *Streptococcus pneumoniae*), and fungal (*Candida albicans*, *Cryptococcus neoformans* and *gattii*) infections of healthy human PBMCs. Whole blood was drawn from six healthy individuals (3 males, 3 females: ages 25–35) through the Duke Healthy Donor Research Protocol, and PBMCs were isolated via a standard Ficoll gradient procedure. Cells were then resuspended in RPMI 5 and plated in duplicate at a concentration of 6×10^6^ cells per well into 24-well plates. Relevant pathogens or controls were then added at different concentrations (influenza viruses at a final concentration of 10^3^ TCID_50_, LPS 1ug/mL, Poly I:C 5ug/mL, *Streptococcus pneumoniae* and *Escherichia coli* at 10^5^ per well, *Candida albicans*, *Cryptococcus neoformans*, and *Cryptococcus gattii* at 10^6^ per well). Bacteria and fungi were heat-killed prior to exposure to human cells to prevent overgrowth in the culture medium. Cells were then incubated at 37° with 5% CO_2_ for 24 h, at which time cells were harvested and underwent centrifuge purification from culture media. Cells were washed and placed in Quiagen RLT lysis buffer per the manufacturer’s instructions. RNA was then extracted and hybridization, and microarray data collection was performed at Expression Analysis (Durham, NC) using the GeneChip® Human Genome U133A 2.0 Array (Affymetrix, Santa Clara, CA).

Similar to the Ramilo and Tsalik datasets, CEL files were imported and processed using the R Bioconductor package readAffy, normalized using gcrma, and lowly expressed probes, defined as detected in less than four samples, and control probes were excluded. Microarray probe identifiers were mapped to Ensembl genes; data was reduced to the subset of probes that mapped to the classifier gene list; and log_2_ transformed. Eighty-nine percent (84/94) of the RNASeq-based classifier genes were present in the microarray dataset, and these were utilized for analysis (Additional file 1: Table S1). The same regularized multinomial modeling, cross-validation procedure, and performance metrics used in the discovery analysis were applied here to estimate the classifier model on a different gene expression platform.

#### Biological pathway analysis

Gene lists were analyzed using the Database for Annotation, Visualization and Integrated Discovery (DAVID, http://www.david.abcc.ncifcrf.gov) [41] to identify significantly enriched pathways. We also applied weighted gene co-expression network analysis (WGCNA) [42, 43] to the discovery dataset (i.e., 11,131 genes in 136 samples). Using these parameters: power parameter = 6; UPGMA clustering; dynamic tree cutting with method = “hyprid”, deepSplit = 2, and minclustersize = 30, we identified 41 clusters (or “modules”). The aggregate expression of all genes assigned to a module can be summarized using PCA, where the 1st principal component (named eigengene) is used as a summary measure of module gene expression. Because each module eigengene can be thought of as the aggregate expression of all of the genes in that module, we can use the eigengene value to test for association with infection type. Each module eigengene was tested for association with Candidemia infection using linear regression. Modules with parameter estimates with a Benjamini-Hochburg adjusted p value <5% were considered statistically significant. Additionally, each module was assessed for enrichment of KEGG and GO pathways using functions goana and kegga available in the R bioconductor package limma. Ensembl gene identifiers were mapped to entrez gene identifiers, and enrichment was assessed for the set of genes within the module compared to all genes that passed quality control and mapped to an entrez gene. Enrichment p values were adjusted for multiple testing within each module using the Benjamini-Hochberg adjustment.

#### Beta-d-glucan testing

Serum samples from all subjects with candidemia, 5 healthy subjects, and 20 subjects with viral infection underwent BDG testing (Viracor Eurofins) (range <31 to >500). Values of >500 were processed as 501, and values <31 were processed as 30. AuROCs were calculated for the BDG test values and the candidemia component of the gene expression signature, separately for the discovery and validation cohorts, restricted to the subset of subjects with both BDG testing and gene expression. BDG and gene expression auROCs were compared using the DeLong test. BDG and gene expression data were also compared by Spearman correlation. Mann-Whitney test was used for the comparison of means.

## Results

### Study population

We enrolled 48 hospitalized adult subjects at the time of first blood culture positivity for *Candida* spp. from 2011 to 2014 at Duke University Medical Center (a minimum of 2 days after initial blood culture collection), along with serial sampling on a subset of patients (Table 1, Fig. 1, Additional file 1: Tables S2 and S3). In addition, we enrolled patients with similar clinical backgrounds but with a proven acute respiratory viral infection, acute bacterial (pneumonia or bacteremia) infection, or clinically adjudicated non-infectious illness, as well as uninfected healthy subjects (n=151, Table 1, Fig. 1, Additional file 1: Tables S4 and S5). The study included subjects from a variety of clinical backgrounds, including solid organ transplants, stem cell transplants, hematologic malignancies, patients in the ICU with central venous catheters, and others. A total of 7 different *Candida* spp. were identified, most commonly *C. albicans* and *C. glabrata*.

**Table 1:** Demographics of the study population

	Bacterial, Viral, & SIRS		Candidemia		***P*** value*
	Discovery (n=100)	Validation (n=21)	Discovery (n=23)	Validation (n=25)	
**Mean age (years) ± SD**	54.0 ± 20.8	43.4 ± 21.3	54.1 ± 17.2	51.8 ± 16.9	0.93
**Solid organ transplant**	4 (4)	0 (0)	5 (22)**	8 (32)	0.0002
**Liver**	0 (0)		1 (14)	1 (13)	
**Heart**	0 (0)		1 (14)	2 (25)	
**Lung**	1 (25)		4 (57)	4 (50)	
**Kidney**	3 (75)		1 (14)	1 (13)	
**Cancer diagnosis**	9 (9)	1 (5)	4 (17)	8 (34)	0.04
**ICU**	18 (18)	6 (27)	11 (48)	6 (26)	0.34
**HIV**	1 (1)	0 (0)	1 (4)	0 (0)	0.67
**30-day mortality**	6 (6)	1 (5)	4 (17)	3 (12)	0.23
**Mean QSOFA ± SD**	1.03 ± 0.93	1.00 ± 1.00	0.75 ± 0.77	0.52 ± 0.67	0.03

Values are presented as n (%) unless otherwise specified

*SD* standard deviation, *ICU* intensive care unit, *HIV* human immunodeficiency virus, *QSOFA* Quick Sequential Organ Failure Assessment

Full demographics were not available on all subjects

*Significance defined as *p*<0.05 for all candidemia vs. all other groups

**7 organ transplants on 5 subjects

### Discovery and validation cohorts

Subjects and controls were divided at random into discovery and validation cohorts for initial analysis. The discovery cohort and validation cohorts included 138 subjects and 61 subjects, respectively (Fig. 1). In the discovery cohort, 23 subjects were adjudicated as having bloodstream infection with *Candida* spp. in the absence of other types of infection. Thirty-five subjects were included with confirmed bacterial infection and 48 with confirmed viral infection (both monomicrobial) as controls. Additionally, as patients may also present clinically with acute non-infectious diseases, we included 17 subjects with acute non-infectious illness, labeled as systemic inflammatory response syndrome (SIRS). In the validation cohort, there were 25 subjects with candidemia, along with 10 subjects with confirmed bacterial infection and 11 subjects with confirmed viral infection (both monomicrobial). Fifteen healthy subjects were also included in each cohort as controls—the mean age of the healthy controls was 20.9 years in the discovery dataset and 33.5 years in the validation dataset. Sixty-five percent of the candidemic subjects in the discovery cohort and 80% in the validation cohort were on antifungal treatment at the time of initial sampling (see cohort data in Additional file 1: Supplementary Methods, and Additional file 2: Figure S1).

### The transcriptional response to candidemia is robust and reveals antifungal defense mechanisms

**Fig. 2:**
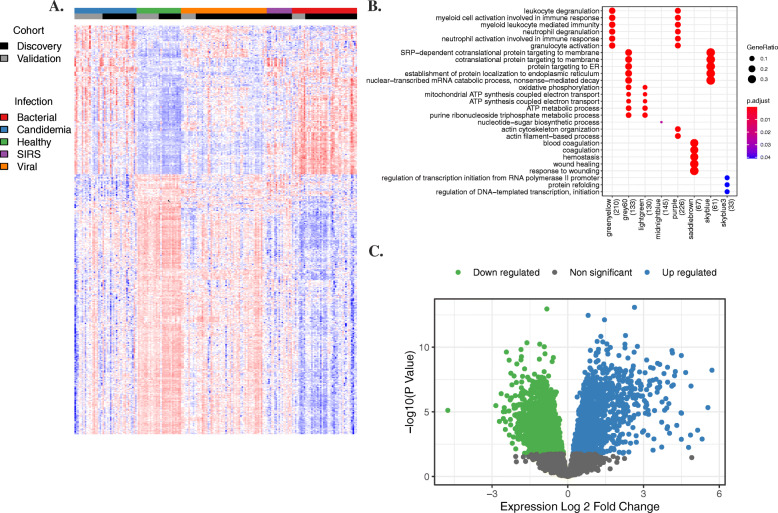
Transcriptional response to candidemia. **A** Heatmap highlighting the differentially expressed genes between patients with candidemia and healthy controls based on combination analysis results including both discovery and validation data, adjusted p value <0.05. **B** Dot-plot demonstrating WGCNA fold enrichment scores. Modules with fold enrichment scores with FDR p value <0.05 were considered significant. **C** Volcano plot demonstrating the differentially expressed genes when comparing candidemia patients and healthy controls

Candidemia triggered a strong transcriptomic response in human hosts with 1641 genes differentially upregulated compared to healthy controls (Fig. 2). These upregulated genes corresponded to known components of the host immune response to fungal infection, including innate immune responses, defense response to fungus, leukocyte migration, and response to yeast. Other stress-associated pathways included response to cytokine, inflammatory response, cellular response to oxidative stress, and host regulation of heme synthesis and iron metabolism. There were 2316 downregulated genes clustered into immune processes such as adaptive immune response, regulation of immune response, B cell proliferation, humoral immune response, immunoglobulin production, and T cell co-stimulation. To further elucidate how transcriptomic responses define active biological pathways in the host, we performed weighted gene co-expression network analysis (WGCNA) [42, 43] to identify clusters of correlated genes associated with candidemia compared to healthy controls (Fig. 2, Additional file 1: Table S6). Clusters significantly upregulated in candidemia included pathways of immune activation and inflammation, including innate immune response and neutrophil activation, migration, and degranulation.

### The transcriptional response to candidemia is unique compared to other infectious triggers

**Fig. 3:**
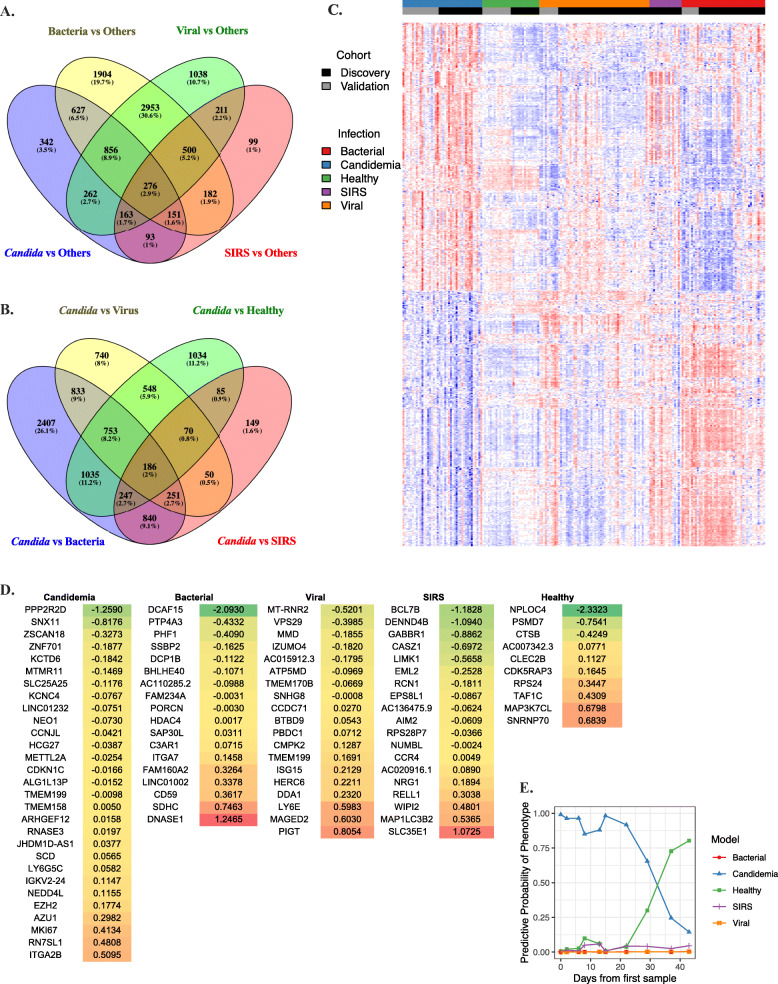
Transcriptional response to candidemia compared to other phenotypes. A* Differentially expressed genes (adj P <0.05) in response to different infection phenotypes. All genes, infection phenotypes compared to all others. B* Differentially expressed genes (adj P <0.05) in response to different infection phenotypes. All genes, *Candida* compared to each other phenotype. **C** Heatmap demonstrating differences in gene expression between infection phenotypes. **D** Genes involved in each phenotype of the multinomial classifier including model coefficients. Colors correspond to coefficient value (green: lower values, red: higher values). **E** Example of predicted probabilities of the specified condition over time. In this case, the subject’s predicted probability of candidemia decreased over time with antifungal treatment whereas the probability of a healthy state increased. *(https://bioinfogp.cnb.csic.es/tools/venny/index.html)

In addition to healthy controls, we also performed univariate comparisons between the transcriptomic responses to candidemia and acute bacterial and viral infection as well as non-infectious SIRS. While there were some conserved components of the host response observed across infection phenotypes, there were also 342 (12%) genes uniquely differentially expressed during candidemia compared to all others (Fig. 3, Additional file 1: Table S7, Additional file 2: Figure S2). This highlights that the transcriptional response to candidemia has unique features compared to other classes of infection. Interestingly, when the transcriptomic response to candidemia was compared to that of other pathogen classes, the top genes upregulated in candidemia again clustered into pathways weighted toward neutrophil activation and heme biosynthesis, further highlighting the strength of these responses during fungal infection (Additional file 1: Table S8).

### A multinomial gene expression classifier distinguishes candidemia from viral or bacterial infection

We next used regularized multinomial logistic regression analyses to determine a set of genes (“signature”) that was most consistently co-regulated across samples from each group of infected subjects. For *Candida* infection, prior work in a mouse model demonstrated that gene expression signatures discriminate early and late invasive candidiasis and that signal intensity decreases over time [26]. Thus, for the development of a diagnostic classifier, we utilized only the first RNA sample obtained for each *Candida* subject after initial blood culture positivity (median 5 days, range 2–23 days). All other acute infection phenotypes only had one RNA sample per subject per episode, taken at the time of initial presentation with their respective infections.

**Fig. 4:**
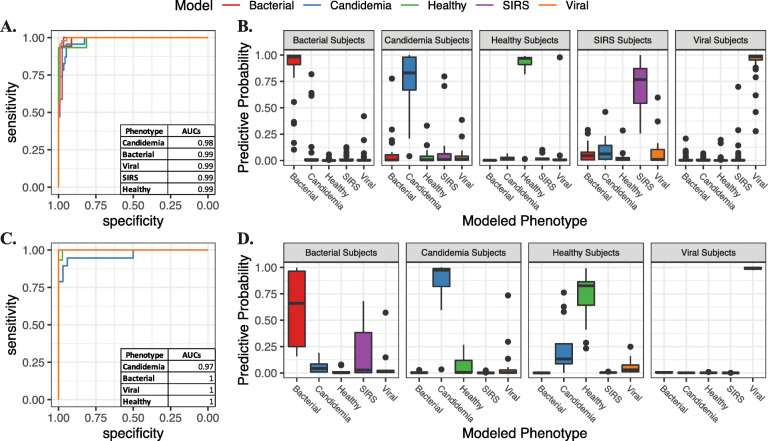
Multinomial gene expression classifier. **A** ROCs of the multinomial classifier performance for each infection phenotype in the discovery cohort. **B** Boxplots demonstrating predictive probability of the classifier for each infection phenotype in the discovery cohort. Infection class as established by the classifier was determined by the phenotype with the highest predictive probability per subject. **C** ROCs of the multinomial classifier performance for each infection phenotype in the validation cohort. **D** Boxplots demonstrating predictive probability of the classifier for each infection phenotype in the validation cohort. Infection class as established by the classifier was determined by the phenotype with the highest predictive probability per subject

Model performance was assessed with auROCs and confusion matrices for all infection classes. All performance measures were cross-validated. We identified a 94-gene classifier that could accurately distinguish candidemia, bacterial, viral, SIRS, and healthy phenotypes (Fig. 3). AuROCs were 0.98 (95%CI 0.96-1) for candidemia, 0.99 (95%CI 0.98-1) for both the bacterial and viral infection, 0.99 (95%CI 0.97-1) for SIRS, and 0.99 (95%CI 0.96-1) for healthy subjects (Fig. 4, Additional file 1: Table S9). On comparison of signature performance between species, there was a small increase in performance with *C. tropicalis* infections (p=0.0446 *tropicalis* vs *albicans*, p=0.0203 *tropicalis* vs *glabrata*, p=0.007 *tropicalis* vs *parapsilosis*), although the differences were quantitatively small and analysis was limited by the small *n* of each subgroup (Additional file 2: Figure S3). Importantly, signature performance did not vary across a number of important clinical variables (total white blood cell count, transplant status, active malignancy, etc. Additional file 1: Table S10, Additional file 2: Figure S4).

The signature derived from the discovery cohort was then used to predict infection class in the validation dataset. Per-class auROCs and confusion matrices were computed. Performance in the validation cohort was equally good: auROCs were 0.97 (95%CI 0.90-1) for candidemia, and 1 for bacterial infection (95%CI 1-1), viral infection (95%CI 1-1), and healthy subjects (95%CI 0.99-1).

### A blood-based gene expression signature of candidemia is maximally expressed at peak illness and decreases in intensity over time

Once a *Candida*-specific diagnostic signature was identified, we sought to examine signal intensity over time as discrimination between early and late disease and defining response to treatment can have an impact on a patient’s clinical care, treatment options, and prognosis. A total of 28 subjects with candidemia had samples collected at more than one date after culture positivity, ranging from 2 to 14 samples per subject. Samples were collected 2 to 80 days from the initial culture. When comparing quantitative levels of expression of genes in the signature for these subjects, we found that the overall trend in signal intensity decreased from the first to the last time-point in subjects with isolated candidemia. However, there was marked variability in quantitative signal strength and time to resolution between subjects. There was an expected inverse correlation seen between quantitative gene expression and days from positive blood culture (ρ = −0.441, p=.0009). In several subjects where appropriate samples were available, the signature-derived predicted probability of candidemia decreased over time with therapy, and eventually, those subjects were predicted by the model to be healthy once candidemia had resolved (Fig. 3E).

### Validation of *Candida* signature in other cohorts

**Fig. 5:**
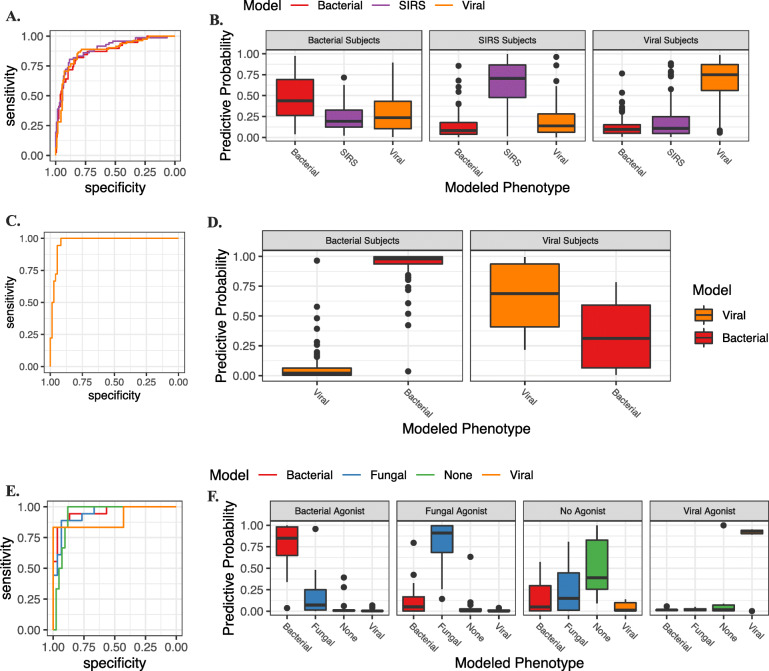
Validation cohorts. ROCs (**A**) and boxplots (**B**) of the multinomial classifier performance for each infection phenotype in the Tsalik et al. cohort. **C** ROCs (**C**) and boxplots (**D**) of the multinomial classifier performance for each infection phenotype in the Ramilo et al. cohort. ROCs (**E**) and boxplots (**F**) of the multinomial classifier performance for each infection phenotype in the in vitro cohort. Infection class as established by the classifier was determined by the phenotype with the highest predictive probability per subject

Given the uniqueness of this dataset and lack of public gene expression data on candidemic subjects, for validation, we next applied the classifier to two independent gene expression data sets from human subjects with acute bacterial and viral illnesses (Ramilo, et al. and Tsalik, et al.) [19, 23] (Fig. 5). When applied to the Ramilo et al. dataset, the novel classifier performed well with an auROC 0.97 (0.95%CI 0.94-1) (Additional file 1: Table S11). When applied to the Tsalik et al. dataset, auROCs were 0.87 (95%CI 0.80–0.93) for bacterial infection, 0.88 for viral (95%CI 0.82–0.92), and 0.89 (95%CI 0.84–0.94) for noninfectious illness (Additional file 1: Table S12).

Next, we compared the candidemia results to gene expression data from an in vitro stimulation assay whereby peripheral blood mononuclear cells (PBMCs) were isolated from healthy individuals and then exposed to pathogens from multiple classes. In this model, cells were then harvested at 24 h post-exposure to analyze transcriptomic responses during experimental viral (influenza), bacterial (*Streptococcus pneumonia* or *Escherichia coli*), and fungal (*Candida albicans* or *Cryptococcus neoformans* or *gattii*) infections. We then applied the human candidemia classifier to these data where it accurately identified the relevant pathogen exposure—auROCs were 0.94 (95%CI 0.88–0.99) for fungal infection, 0.96 (95%CI 0.89-1) for bacterial, 0.90 (95%CI 0.69-1) for viral infection, and 0.94 (95%CI 0.86-0.99) for healthy control cells (Fig. 5, Additional file 1: Table S13). To further clarify the distinction in signature performance between *Candida* and *Cryptococcus*, we examined the predictive probabilities and confusion matrix at the agonist level. We observed that there was not a statistically significant difference between *Candida* and *Cryptococcus* (ANOVA F test p value = 0.2866).

### Comparison to BDG

We next sought to compare the diagnostic accuracy of serum BDG levels with the novel transcriptomic biomarker signature. The mean level of BDG at the time of first blood culture positivity for candidemia was 246 pg/mL ± 192 (range <31 to >500), which was not significantly higher than the mean for last BDG at 235 pg/mL ± 189 (range <31 to >500, p=0.85). Serial BDG measurements showed that only 43% (13/30) of subjects had decreasing values of BDG in response to treatment, and the rate of decrease was highly variable. The overall BDG auROC was 0.90 (95%CI 0.80–.97). When broken down into discovery and validation cohorts, the candidemia component of the gene expression classifier had higher performance characteristics than BDG though this result was not statistically significant. The discovery auROC for gene expression was 1 (95%CI 1-1) compared to 0.98 (95%CI 0.94-1) for BDG (p=0.39), the validation auROC was 0.94 (95%CI 0.81-1) for gene expression compared to 0.83 (95%CI 0.63-0.97) for BDG (p=0.35). BDG level was found to be moderately inversely correlated with days from positive blood culture (ρ = −0.29, p=0.05) and mildly correlated with quantitative gene expression (ρ = 0.258, p=0.084).

## Discussion

Multiple pathogen-based diagnostic modalities for candidemia are currently available but often hindered by delayed time-to-result and/or suboptimal sensitivity and specificity [11, 12, 14, 15]. Host-derived biomarker approaches offer the potential to fill critical diagnostic niches, including rapid (even point-of-care) detection of multiple pathogen classes at once, and improved specificity through identification of pathologic host responses. In this work, we have for the first time defined the host response to candidemia as seen through the lens of the transcriptome in circulating leukocytes. This has enabled the development of a host signature able to differentiate acute fungal infection from viral, bacterial, and SIRS phenotypes that may also cause similar acute illness in at-risk hosts.

The host response to *Candida* infection has both shared and unique features compared to other pathogen classes, and this is manifested at the transcriptional level in the peripheral blood. We found over 1600 upregulated genes in the presence of candidemia compared to healthy controls. Many of these genes reflected known components of the immune response to fungal infection or critical illness including cytokine signaling, inflammatory responses, and cellular responses to oxidative stress. Some, like neutrophil activation and migration, are known to play a role in antifungal defense, but the strength of these responses, even when compared to similarly ill subjects with acute bacterial infections, was surprising and highlights the critical importance of these pathways in clearing *Candida* spp. Other enriched pathways identify potentially novel host response mechanisms to *Candida* infection such as alterations in the regulation of heme synthesis. While iron is known to be critical for fungal pathogens such as *Candida *in vitro [44], our results suggest the human host may manipulate this system as part of the response to fungal infection.

Through multinomial logistic regression analyses, we identified a unifying signature that could model the host response to multiple different illness etiologies at once with a high degree of accuracy (auROC 0.98 for candidemia). The candidemia component of this classifier performed better than the standard of care diagnostic BDG test. Importantly, a strength of the candidemia signature is that it exhibited robust performance despite over 70% of the cohort being on active empiric antifungal treatment at the time of initial testing, a common clinical approach that impairs many traditional pathogen detection strategies such as blood culture. Furthermore, the *Candida* classifier performs well across a wide array of typical clinical backgrounds including neutropenia and multiple types of immunosuppression, as well as across 7 different *Candida* species. Another advantage to the multinomial approach presented here is that a single test can inform the diagnosis of multiple conditions (i.e., candidal, bacterial, viral, SIRS, healthy) simultaneously. One limitation of this study is that while the in silico and in vitro validation data support generalizability, this was a single-center study and will require validation in other candidemic populations once additional cohorts/datasets are available. While the cohort is diverse, the relatively small candidemia sample size limits sub-group analysis, and further work with larger groups of neutropenic and other types of immunocompromised patients will be necessary. Additionally, the study design limits our ability to identify test performance at earlier times during *Candida* infection where treatment may be most efficacious, as subjects were not enrolled until their blood cultures had turned positive. However, during in vitro infections, a marked transcriptomic response matching that seen in patients with proven candidemia was seen within 24 h of exposure to fungal organisms, suggesting that the transcriptomic signature of candidemia is likely to be present at much earlier times than we have been able to demonstrate in human subjects. Additionally, while the host response to *Candida* involves components that typify the response to many fungal organisms, understanding how such a signature may perform in other fungal diseases such as invasive mold infections will require further study. Finally, this study did not directly evaluate the performance of the signature in cases of invasive candidiasis (esophageal, abdominal, etc.) without candidemia, so the signal strength and efficacy in these infections will need to be formally explored.

## Conclusions

The host response to candidemia in hospitalized adults is highly conserved and is distinct from the transcriptomic responses to acute viral and bacterial infection. Clinic-ready platforms capable of operationalizing PCR-based signatures of the sizes demonstrated herein already exist, offering a proximal pathway to clinical application of these findings. Harnessing these pathogen class-specific responses allows for a better understanding of the immunopathogenesis of fungal infections in human hosts and shows promise for the development of host gene expression-based assays to simultaneously differentiate multiple types of clinical illnesses in acutely ill patients.

## Supplementary Information

**Additional file 1: Supplementary Methods and Supplementary Tables S1-S13**. **Table S1**. Genes Without Microarray Probes. RNASeq-based classifier genes present in the microarray dataset. **Table S2**. Additional Demographics of Candidemic Subjects. Additional demographic information on all candidemic subjects. **Table S3**. Clinical Information on Subjects with Candidemia. Additional clinical information on all candidemic subjects. **Table S4**. Comparator Phenotypes – Discovery Cohort. Responsible etiologies/pathogens for all bacterial, viral, and SIRS comparator phenotypes included in the discovery cohort of the analysis. **Table S5**. Comparator Phenotypes – Validation Cohort. Responsible pathogens for all bacterial and viral comparator phenotypes included in the validation cohort of the analysis. **Table S6**. Significant biological process modules by WGCNA, *Candida* vs. Healthy. Biological process modules by WGCNA, divided by cluster. **Table S7.** Unique genes, *Candida* vs other pathogen classes. Genes uniquely differentially expressed during candidemia compared to all others. **Table S8**. Top functional annotation clusters, *Candida* vs other pathogen classes. Top 5 functional annotation clusters, Candida vs other pathogen classes, sorted by descending enrichment score. **Table S9**. Confusion matrices for the multinomial classifier. Confusion matrices for the multinomial classifier, demonstrating high test accuracy for all studied infection types and healthy controls. **Table S10**. Signature performance is not associated with important clinical variables. Correlation of clinical variables and signature performance. **Table S11.** Ramilo, *et al.* Validation. Confusion matrix for classifier performance in the Ramilo, et al. validation dataset. **Table S12.** Tsalik, *et al.* Validation. Confusion matrix for classifier performance in the Tsalik, et al. validation dataset. **Table S13**. PBMC Validation. Confusion matrix for classifier performance in the PBMC validation dataset.

**Additional file 2: Supplementary Figures S1-S4. Figure S1**. Boxplot of the candidemia model predictive probabilities in the discovery and validation cohort by anti-fungal treatment. **Figure S2**. Differentially expressed genes in response to different infection phenotypes. **Figure S3.** Predictive Probability of Candidemia by *Candida* species. **Figure S4**. Signature performance with demographics included in the model.

## Data Availability

Gene expression data generated in this study have been deposited in the NCBI Gene Expression Omnibus (GEO) with the following accession number: GSE176262 (Discovery and Validation datasets; https://www.ncbi.nlm.nih.gov/geo/query/acc.cgi?acc=GSE176262) [33]. This study further used published datasets GEO GSE6269 (Ramilo, et al. [23]; https://www.ncbi.nlm.nih.gov/geo/query/acc.cgi?acc=GSE6269) [39]; and GEO GSE63990 (Tsalik, et al. [19]; https://www.ncbi.nlm.nih.gov/geo/query/acc.cgi?acc=GSE63990) [41]. The algorithm and sample code to reproduce the prediction model development are included in Additional file 1: Supplementary Methods.
