# Supplementary material for: The host transcriptional response to Candidemia is dominated by neutrophil activation and heme biosynthesis and supports novel diagnostic approaches
Source: Genome Med. 2021 Jul 5;13:108. doi: 10.1186/s13073-021-00924-9 (PMC8259367; doi:10.1186/s13073-021-00924-9)
Supplement: Supplementary file 1 — Additional file 1: Supplementary Methods and Supplementary Tables S1-S13. Table S1. Genes Without Microarray Probes. RNASeq-based classifier genes present in the microarray dataset. Table S2. Additional Demographics of Candidemic Subjects. Additional demographic information on all candidemic subjects. Table S3. Clinical Information on Subjects with Candidemia. Additional clinical information on all candidemic subjects. Table S4. Comparator Phenotypes – Discovery Cohort. Responsible etiologies/pathogens for all bacterial, viral, and SIRS comparator phenotypes included in the discovery cohort of the analysis. Table S5. Comparator Phenotypes – Validation Cohort. Responsible pathogens for all bacterial and viral comparator phenotypes included in the validation cohort of the analysis. Table S6. Significant biological process modules by WGCNA, Candida vs. Healthy. Biological process modules by WGCNA, divided by cluster. Table S7. Unique genes, Candida vs other pathogen classes. Genes uniquely differentially expressed during candidemia compared to all others. Table S8. Top functional annotation clusters, Candida vs other pathogen classes. Top 5 functional annotation clusters, Candida vs other pathogen classes, sorted by descending enrichment score. Table S9. Confusion matrices for the multinomial classifier. Confusion matrices for the multinomial classifier, demonstrating high test accuracy for all studied infection types and healthy controls. Table S10. Signature performance is not associated with important clinical variables. Correlation of clinical variables and signature performance. Table S11. Ramilo, et al. Validation. Confusion matrix for classifier performance in the Ramilo, et al. validation dataset. Table S12. Tsalik, et al. Validation. Confusion matrix for classifier performance in the Tsalik, et al. validation dataset. Table S13. PBMC Validation. Confusion matrix for classifier performance in the PBMC validation dataset. [file 13073_2021_924_MOESM1_ESM.docx]

**Supplementary Methods and Supplementary Tables**

**Supplementary Methods**

Algorithm and Code for Classifier Development

The R package glmnet was used to develop the classification model. The R syntax to generate the model is as follows:

# XX = t(cpm(NormalizedCounts, log=T)), rows are samples, columns are transcripts

# key = vector of phenotype – candidemia, viral, etc.

# 10-fold cross validation to optimize lambda:

Lmo_cv<- cv.glment(XX, key, alpha = 1, standardize = TURE, family = “multinomial”, nfolds = 10, type.measure = ‘mse’)

# model using the optimallambda:

Lmo = glmnet(XX, key, alpha = 1, standardize =TRUE, family=”multinomial”, lambda = lmo_cv$lambda.min)

To assess performance using leave one out cross validation:

1. For i in 1: number of rows in XX:
   1. Generate lmo_cv using XX[-i,] and key[-i]
   2. Generate lmo using XX[-i,] and key[-i], and lmo_cv$lambda.min
   3. Predict phenotype in ith sample using syntax:
      1. predict(lmo, XX[i,], type = “response”)
2. For each phenotype, use pROC::roc to calculate rocAUC
   1. True phenotype: does key == phenotype of interest yes/no (e.g. is Candidemia yes/no or is Viral yes/no)
   2. Prediction: the predicted probabilities estimated in 1c for the phenotype of interest

Cohort Data

In the discovery cohort, 15 of 23 subjects were on systemic antifungals at the time of first sample collection (mean duration of antifungals prior to sample collection of 6.11 days). One subject was started on antifungals that same day, although it is unknown if a dose was received prior to sample collection. Seven subjects had limited data available in the electronic health record, thus the date of antifungal initiation was unable to be determined. No subjects were confirmed to be off of systemic antifungals at the time of first sample collection. Initial and final antifungals are listed in Table S2. In the validation cohort, 20 of 25 subjects were on systemic antifungals at the time of first sample collection (mean duration of antifungals prior to sample collection of 5.13 days). Three subjects were started on antifungals that same day. Two subjects had limited data available in the electronic health record, thus the data of antifungal initiation was unable to be determined. One subject was not on systemic antifungals at the time of sample collection. Overall, the transcriptomic signal of candidemia was sufficient for detection and diagnostic performance despite the presence of empiric antifungal treatment.

**Supplementary Tables**

**Table S1. Genes Without Microarray Probes**

| **Gene** |
| --- |
| ENSG00000241294 |
| ENSG00000274213 |
| ENSG00000262877 |
| ENSG00000270972 |
| ENSG00000253981 |
| ENSG00000210082 |
| ENSG00000280734 |
| ENSG00000267519 |
| ENSG00000227097 |
| ENSG00000282508 |

**Table S2. Additional Demographics of Candidemic Subjects**

| Clinical Variable | Candidemia Discovery Cohort (n=23) | Candidemia Validation Cohort (n=25) |
| --- | --- | --- |
| Gender (female) | 11 (48) | 12 (48) |
| Ethnicity  Hispanic/Latino  Nonhispanic/Latino | 2 (9)  21 (91) | 0 (0)  25 (100) |
| Race  American Indian/Alaska Native  Native Hawaiian/Pacific Islander  Asian  White  Black/African American  Multiracial  Unknown | 0 (0)  0 (0)  0 (0)  14 (61)  7 (30)  2 (9)  0 (0) | 1 (4)  0 (0)  0 (0)  18 (72)  6 (24)  0 (0)  0 (0) |
| Vasopressor requirement | 2 (9) | 5 (20) |
| Diabetes mellitus | 10 (43) | 11 (44) |
| Post-surgical | 9 (39) | 10 (40) |
| TPN | 1 (4) | 4 (16) |
| Indwelling central line | 17 (74) | 22 (8) |
| IVDU | 1 (4) | 2 (8) |
| Hemodialysis | 4 (17) | 1 (4) |
| Time from transplant  <6 months  6 months to <1 year  1-3 years  >3 years | 5 (71)  0 (0)  0 (0)  2 (29) | 4 (50)  0 (0)  1 (13)  3 (38) |
| Type of Transplant*  Liver  Heart  Lung  Kidney  Small bowel | 1 (14)  1 (14)  4 (57)  1 (14)  0 (0) | 1 (13)  2 (25)  4 (50)  1 (13)  0 (0) |
| Treated for rejection in last 90 days | 0 (0) | 0 (0) |
| Stem cell transplant | 0 (0) | 2 (8) |
| Hematologic malignancy | 2 (9) | 5 (20) |
| Neutropenic** | 3 (13) | 6 (24) |

Values are reported as number (%)

* Number refers to the number of transplants and not the number of subjects.

** Neutropenia is defined as a neutrophil count < 1500 cells/µl.

**Table S3. Clinical information on Candidemic Subjects**

| Clinical Manifestations, Labs, and Treatment | Candidemia Discovery Cohort  (n=23) | Candidemia Validation Cohort (n=25) | P value |
| --- | --- | --- | --- |
| Additional sites of infection  Eyes  Heart  Hepatosplenic  Peritonitis  Esophagus  CNS  Lungs (empyema)  Genitourinary  Soft tissue  Bone  None  Unknown | 1  1  0  0  0  0  3  0  0  0  13  5 | 1  0  0  1  2  0  4  2  0  0  15  0 |  |
| *Candida* spp.*  *C. albicans*  *C. glabrata*  *C. parapsilosis*  *C. tropicalis*  *C. krusei*  *C. dubliniensis*  *C. zeylanoides* | 9  7  5  2  1  0  0 | 4  7  3  9  2  1  1 |  |
| Initial Antifungal  Fluconazole  Micafungin  Voriconazole  Isavuconazole  Posaconazole  Amphotericin | 9  12  0  0  0  2 | 2  22  0  0  0  1 |  |
| Final Antifungal  Fluconazole  Micafungin  Voriconazole  Isavuconazole  Posaconazole  Amphotericin  Combination therapy  Unknown | 8  7  0  0  0  1  3  4 | 10  13  0  0  0  1  1  0 |  |
| Number of hospitalized days pre-dx (mean ± SD) | 11.94 ± 13.94 days (range 0-50) | 12.60 ± 17.55 days (range 0-75) | p=0.73 |
| Total duration of hospitalization (mean ± SD) | 41.39 ± 51.50 days (range 5-221) | 28.32 ± 28.13 days (range 4-109) | p=0.32 |
| Fever at time of Dx** | 10 | 15 |  |
| Hypothermia at time of Dx | 1 | 0 |  |

*Two subjects had simultaneous infection with more than one *Candida* species.

**Nine subjects had limited medical records, and temperature was not recorded.

**Table S4. Comparator Phenotypes – Discovery Cohort**

| **Phenotype** | **Pathogen (n)** |
| --- | --- |
| Bacterial | *Streptococcus pneumoniae* (10) |
| Bacterial | *Staphylococcus aureus* (7) |
| Bacterial | Polymicrobial (6) |
| Bacterial | *Escherichia coli* (5) |
| Bacterial | *Haemophilus influenza* (2) |
| Bacterial | *Klebsiella pneumoniae* (2) |
| Bacterial | *Legionella* sp. (1) |
| Bacterial | *Streptococcus agalactiae* (1) |
| Bacterial | *Streptococcus pyogenes* (1) |
| Viral | Influenza A (20) |
| Viral | Influenza B (6) |
| Viral | Rhinovirus (6) |
| Viral | Respiratory syncytial virus (5) |
| Viral | Human metapneumovirus (4) |
| Viral | Coronavirus (3) |
| Viral | Coxsackievirus/Echovirus (3) |
| Viral | Parainfluenza (1) |
| SIRS | Heart failure (5) |
| SIRS | Pulmonary edema (2) |
| SIRS | Gastrointestinal bleed (1) |
| SIRS | Gout, venous stasis (1) |
| SIRS | Heart failure and arrythmia (1) |
| SIRS | Hemochromatosis (1) |
| SIRS | Heroin overdose (1) |
| SIRS | Hypertensive emergency (1) |
| SIRS | Lung cancer with hemoptysis (1) |
| SIRS | Nephrolithiasis (1) |
| SIRS | Pulmonary embolus and pulmonary arterial hypertension (1) |
| SIRS | Tracheobronchomalacia (1) |

**Table S5. Comparator Phenotypes – Validation Cohort**

| **Phenotype** | **Pathogen (n)** |
| --- | --- |
| Bacterial | *Streptococcus pneumoniae* (5) |
| Bacterial | *Staphylococcus aureus* (2) |
| Bacterial | *Escherichia coli* (1) |
| Bacterial | *Proteus mirabilis* (1) |
| Bacterial | *Streptococcus pyogenes* (1) |
| Viral | Influenza A (10) |
| Viral | Rhinovirus (1) |

**Table S6. Significant biological process modules by WGCNA, *Candida* vs. Healthy**

| **Cluster** | **ID** | **Description** | **Adjusted p value** | **Count** |
| --- | --- | --- | --- | --- |
| *cyan* | GO:0022900 | electron transport chain | 0.00722622 | 12 |
| *cyan* | GO:0006119 | oxidative phosphorylation | 0.01211998 | 10 |
| *cyan* | GO:0010499 | proteasomal ubiquitin-independent protein catabolic process | 0.01211998 | 5 |
| *cyan* | GO:0045333 | cellular respiration | 0.01387226 | 11 |
| *cyan* | GO:0042775 | mitochondrial ATP synthesis coupled electron transport | 0.01387226 | 8 |
| *cyan* | GO:0042773 | ATP synthesis coupled electron transport | 0.01387226 | 8 |
| *cyan* | GO:0015980 | energy derivation by oxidation of organic compounds | 0.02774203 | 12 |
| *cyan* | GO:0022904 | respiratory electron transport chain | 0.02774203 | 8 |
| *cyan* | GO:0009060 | aerobic respiration | 0.02774203 | 7 |
| *cyan* | GO:0006091 | generation of precursor metabolites and energy | 0.02802918 | 16 |
| *cyan* | GO:0009141 | nucleoside triphosphate metabolic process | 0.03248529 | 13 |
| *darkolivegreen* | GO:0000209 | protein polyubiquitination | 0.0105159 | 8 |
| *darkolivegreen* | GO:0006511 | ubiquitin-dependent protein catabolic process | 0.0105159 | 11 |
| *darkolivegreen* | GO:0019941 | modification-dependent protein catabolic process | 0.0105159 | 11 |
| *darkolivegreen* | GO:0043632 | modification-dependent macromolecule catabolic process | 0.0105159 | 11 |
| *darkolivegreen* | GO:0043161 | proteasome-mediated ubiquitin-dependent protein catabolic process | 0.01058071 | 9 |
| *darkolivegreen* | GO:0070936 | protein K48-linked ubiquitination | 0.01971656 | 4 |
| *darkolivegreen* | GO:0010498 | proteasomal protein catabolic process | 0.01971656 | 9 |
| *green* | GO:0042744 | hydrogen peroxide catabolic process | 4.75E-06 | 10 |
| *green* | GO:0051187 | cofactor catabolic process | 6.29E-06 | 12 |
| *green* | GO:0042743 | hydrogen peroxide metabolic process | 6.56E-06 | 12 |
| *green* | GO:0042737 | drug catabolic process | 0.00011176 | 15 |
| *green* | GO:0017001 | antibiotic catabolic process | 0.00077286 | 10 |
| *green* | GO:0051186 | cofactor metabolic process | 0.00077286 | 39 |
| *green* | GO:0006778 | porphyrin-containing compound metabolic process | 0.00202378 | 9 |
| *green* | GO:0015893 | drug transport | 0.00202378 | 15 |
| *green* | GO:0015701 | bicarbonate transport | 0.00361917 | 7 |
| *green* | GO:0016999 | antibiotic metabolic process | 0.00528713 | 15 |
| *green* | GO:0033013 | tetrapyrrole metabolic process | 0.00823644 | 10 |
| *green* | GO:0020027 | hemoglobin metabolic process | 0.01315437 | 5 |
| *green* | GO:0006779 | porphyrin-containing compound biosynthetic process | 0.01988423 | 7 |
| *green* | GO:0046501 | protoporphyrinogen IX metabolic process | 0.01988423 | 5 |
| *green* | GO:0006820 | anion transport | 0.02180041 | 28 |
| *green* | GO:0042168 | heme metabolic process | 0.02180041 | 7 |
| *green* | GO:0048821 | erythrocyte development | 0.02180041 | 7 |
| *green* | GO:0098869 | cellular oxidant detoxification | 0.02270385 | 11 |
| *green* | GO:0033014 | tetrapyrrole biosynthetic process | 0.03173847 | 7 |
| *green* | GO:1990748 | cellular detoxification | 0.03173847 | 11 |
| *green* | GO:0006783 | heme biosynthetic process | 0.04968208 | 6 |
| *greenyellow* | GO:0043299 | leukocyte degranulation | 1.11E-06 | 34 |
| *greenyellow* | GO:0002275 | myeloid cell activation involved in immune response | 1.11E-06 | 34 |
| *greenyellow* | GO:0002444 | myeloid leukocyte mediated immunity | 1.11E-06 | 34 |
| *greenyellow* | GO:0043312 | neutrophil degranulation | 1.11E-06 | 32 |
| *greenyellow* | GO:0002283 | neutrophil activation involved in immune response | 1.11E-06 | 32 |
| *greenyellow* | GO:0002446 | neutrophil mediated immunity | 1.18E-06 | 32 |
| *greenyellow* | GO:0042119 | neutrophil activation | 1.18E-06 | 32 |
| *greenyellow* | GO:0036230 | granulocyte activation | 1.44E-06 | 32 |
| *greenyellow* | GO:0060099 | regulation of phagocytosis, engulfment | 0.02403502 | 4 |
| *greenyellow* | GO:1905153 | regulation of membrane invagination | 0.03187756 | 4 |
| *grey60* | GO:0006614 | SRP-dependent cotranslational protein targeting to membrane | 5.52E-24 | 26 |
| *grey60* | GO:0006613 | cotranslational protein targeting to membrane | 9.27E-24 | 26 |
| *grey60* | GO:0045047 | protein targeting to ER | 5.88E-23 | 26 |
| *grey60* | GO:0072599 | establishment of protein localization to endoplasmic reticulum | 9.74E-23 | 26 |
| *grey60* | GO:0000184 | nuclear-transcribed mRNA catabolic process, nonsense-mediated decay | 5.71E-22 | 26 |
| *grey60* | GO:0070972 | protein localization to endoplasmic reticulum | 5.75E-21 | 26 |
| *grey60* | GO:0000956 | nuclear-transcribed mRNA catabolic process | 2.09E-18 | 28 |
| *grey60* | GO:0006612 | protein targeting to membrane | 2.90E-18 | 26 |
| *grey60* | GO:0006413 | translational initiation | 1.93E-17 | 26 |
| *grey60* | GO:0006402 | mRNA catabolic process | 2.15E-15 | 29 |
| *grey60* | GO:0006605 | protein targeting | 9.26E-15 | 31 |
| *grey60* | GO:0072594 | establishment of protein localization to organelle | 1.83E-14 | 34 |
| *grey60* | GO:0006401 | RNA catabolic process | 2.05E-14 | 29 |
| *grey60* | GO:0090150 | establishment of protein localization to membrane | 1.46E-13 | 26 |
| *grey60* | GO:0046700 | heterocycle catabolic process | 5.91E-12 | 32 |
| *grey60* | GO:0044270 | cellular nitrogen compound catabolic process | 5.91E-12 | 32 |
| *grey60* | GO:0019439 | aromatic compound catabolic process | 7.90E-12 | 32 |
| *grey60* | GO:0034655 | nucleobase-containing compound catabolic process | 9.70E-12 | 31 |
| *grey60* | GO:1901361 | organic cyclic compound catabolic process | 1.33E-11 | 32 |
| *grey60* | GO:0002181 | cytoplasmic translation | 2.01E-11 | 15 |
| *grey60* | GO:0006119 | oxidative phosphorylation | 1.35E-10 | 16 |
| *grey60* | GO:0072657 | protein localization to membrane | 2.25E-09 | 27 |
| *grey60* | GO:0042775 | mitochondrial ATP synthesis coupled electron transport | 4.67E-07 | 11 |
| *grey60* | GO:0042773 | ATP synthesis coupled electron transport | 5.17E-07 | 11 |
| *grey60* | GO:0022900 | electron transport chain | 5.40E-07 | 14 |
| *grey60* | GO:0046034 | ATP metabolic process | 8.40E-07 | 16 |
| *grey60* | GO:0022904 | respiratory electron transport chain | 2.22E-06 | 11 |
| *grey60* | GO:0009205 | purine ribonucleoside triphosphate metabolic process | 3.11E-06 | 16 |
| *grey60* | GO:0009126 | purine nucleoside monophosphate metabolic process | 3.99E-06 | 16 |
| *grey60* | GO:0009167 | purine ribonucleoside monophosphate metabolic process | 3.99E-06 | 16 |
| *grey60* | GO:0009144 | purine nucleoside triphosphate metabolic process | 3.99E-06 | 16 |
| *grey60* | GO:0009199 | ribonucleoside triphosphate metabolic process | 3.99E-06 | 16 |
| *grey60* | GO:0009161 | ribonucleoside monophosphate metabolic process | 7.58E-06 | 16 |
| *grey60* | GO:0022613 | ribonucleoprotein complex biogenesis | 8.01E-06 | 21 |
| *grey60* | GO:0006091 | generation of precursor metabolites and energy | 8.81E-06 | 19 |
| *grey60* | GO:0009141 | nucleoside triphosphate metabolic process | 9.31E-06 | 16 |
| *grey60* | GO:0009150 | purine ribonucleotide metabolic process | 1.08E-05 | 19 |
| *grey60* | GO:0072521 | purine-containing compound metabolic process | 1.08E-05 | 20 |
| *grey60* | GO:0009123 | nucleoside monophosphate metabolic process | 1.51E-05 | 16 |
| *grey60* | GO:0006364 | rRNA processing | 1.86E-05 | 14 |
| *grey60* | GO:0006163 | purine nucleotide metabolic process | 1.89E-05 | 19 |
| *grey60* | GO:0009259 | ribonucleotide metabolic process | 1.89E-05 | 19 |
| *grey60* | GO:0042254 | ribosome biogenesis | 2.22E-05 | 16 |
| *grey60* | GO:0019693 | ribose phosphate metabolic process | 3.40E-05 | 19 |
| *grey60* | GO:0034660 | ncRNA metabolic process | 8.51E-05 | 21 |
| *grey60* | GO:0016072 | rRNA metabolic process | 9.23E-05 | 14 |
| *grey60* | GO:0006120 | mitochondrial electron transport, NADH to ubiquinone | 9.23E-05 | 7 |
| *grey60* | GO:0034470 | ncRNA processing | 0.00012069 | 17 |
| *grey60* | GO:0009117 | nucleotide metabolic process | 0.0001904 | 20 |
| *grey60* | GO:0045333 | cellular respiration | 0.00019064 | 11 |
| *grey60* | GO:0006753 | nucleoside phosphate metabolic process | 0.00020179 | 20 |
| *grey60* | GO:0017144 | drug metabolic process | 0.00033933 | 20 |
| *grey60* | GO:1902600 | proton transmembrane transport | 0.00044897 | 9 |
| *grey60* | GO:0042274 | ribosomal small subunit biogenesis | 0.00051194 | 7 |
| *grey60* | GO:1901798 | positive regulation of signal transduction by p53 class mediator | 0.00094981 | 4 |
| *grey60* | GO:0042776 | mitochondrial ATP synthesis coupled proton transport | 0.00214558 | 4 |
| *grey60* | GO:1990542 | mitochondrial transmembrane transport | 0.0025432 | 7 |
| *grey60* | GO:0015980 | energy derivation by oxidation of organic compounds | 0.00271246 | 11 |
| *grey60* | GO:0042255 | ribosome assembly | 0.00333008 | 6 |
| *grey60* | GO:0072332 | intrinsic apoptotic signaling pathway by p53 class mediator | 0.00333008 | 6 |
| *grey60* | GO:0022618 | ribonucleoprotein complex assembly | 0.00418307 | 11 |
| *grey60* | GO:0015985 | energy coupled proton transport, down electrochemical gradient | 0.00451701 | 4 |
| *grey60* | GO:0015986 | ATP synthesis coupled proton transport | 0.00451701 | 4 |
| *grey60* | GO:1902253 | regulation of intrinsic apoptotic signaling pathway by p53 class mediator | 0.00451701 | 4 |
| *grey60* | GO:0071826 | ribonucleoprotein complex subunit organization | 0.00641089 | 11 |
| *grey60* | GO:2000059 | negative regulation of ubiquitin-dependent protein catabolic process | 0.00679792 | 5 |
| *grey60* | GO:0042273 | ribosomal large subunit biogenesis | 0.00683351 | 6 |
| *grey60* | GO:2001244 | positive regulation of intrinsic apoptotic signaling pathway | 0.00915839 | 5 |
| *grey60* | GO:0006122 | mitochondrial electron transport, ubiquinol to cytochrome c | 0.01198653 | 3 |
| *grey60* | GO:0051444 | negative regulation of ubiquitin-protein transferase activity | 0.01198653 | 3 |
| *grey60* | GO:0042407 | cristae formation | 0.01917344 | 4 |
| *grey60* | GO:0000028 | ribosomal small subunit assembly | 0.02335964 | 3 |
| *grey60* | GO:0007005 | mitochondrion organization | 0.0262639 | 15 |
| *grey60* | GO:0010257 | NADH dehydrogenase complex assembly | 0.0262639 | 5 |
| *grey60* | GO:0032981 | mitochondrial respiratory chain complex I assembly | 0.0262639 | 5 |
| *grey60* | GO:1903051 | negative regulation of proteolysis involved in cellular protein catabolic process | 0.0262639 | 5 |
| *grey60* | GO:0030150 | protein import into mitochondrial matrix | 0.0262639 | 3 |
| *grey60* | GO:1904666 | regulation of ubiquitin protein ligase activity | 0.0262639 | 3 |
| *grey60* | GO:2001242 | regulation of intrinsic apoptotic signaling pathway | 0.03907673 | 7 |
| *grey60* | GO:0006839 | mitochondrial transport | 0.03907673 | 9 |
| *grey60* | GO:0008380 | RNA splicing | 0.04229108 | 13 |
| *grey60* | GO:0007007 | inner mitochondrial membrane organization | 0.04788078 | 4 |
| *grey60* | GO:0072331 | signal transduction by p53 class mediator | 0.04828468 | 8 |
| *grey60* | GO:1903363 | negative regulation of cellular protein catabolic process | 0.04874464 | 5 |
| *grey60* | GO:1901796 | regulation of signal transduction by p53 class mediator | 0.04931535 | 6 |
| *lightgreen* | GO:0006119 | oxidative phosphorylation | 0.0004501 | 11 |
| *lightgreen* | GO:0046034 | ATP metabolic process | 0.0004501 | 14 |
| *lightgreen* | GO:0042775 | mitochondrial ATP synthesis coupled electron transport | 0.0004501 | 9 |
| *lightgreen* | GO:0042773 | ATP synthesis coupled electron transport | 0.0004501 | 9 |
| *lightgreen* | GO:0009205 | purine ribonucleoside triphosphate metabolic process | 0.00045745 | 14 |
| *lightgreen* | GO:0009126 | purine nucleoside monophosphate metabolic process | 0.00045745 | 14 |
| *lightgreen* | GO:0009167 | purine ribonucleoside monophosphate metabolic process | 0.00045745 | 14 |
| *lightgreen* | GO:0009144 | purine nucleoside triphosphate metabolic process | 0.00045745 | 14 |
| *lightgreen* | GO:0009199 | ribonucleoside triphosphate metabolic process | 0.00045745 | 14 |
| *lightgreen* | GO:0022904 | respiratory electron transport chain | 0.00062742 | 9 |
| *lightgreen* | GO:0009161 | ribonucleoside monophosphate metabolic process | 0.00066246 | 14 |
| *lightgreen* | GO:0009141 | nucleoside triphosphate metabolic process | 0.0007784 | 14 |
| *lightgreen* | GO:0009123 | nucleoside monophosphate metabolic process | 0.00115777 | 14 |
| *lightgreen* | GO:0022900 | electron transport chain | 0.00300289 | 10 |
| *lightgreen* | GO:0045333 | cellular respiration | 0.00395754 | 10 |
| *lightgreen* | GO:0006120 | mitochondrial electron transport, NADH to ubiquinone | 0.00395754 | 6 |
| *lightgreen* | GO:0006091 | generation of precursor metabolites and energy | 0.00606789 | 15 |
| *lightgreen* | GO:0009150 | purine ribonucleotide metabolic process | 0.00702608 | 15 |
| *lightgreen* | GO:0015980 | energy derivation by oxidation of organic compounds | 0.00851607 | 11 |
| *lightgreen* | GO:0006163 | purine nucleotide metabolic process | 0.00981269 | 15 |
| *lightgreen* | GO:0009259 | ribonucleotide metabolic process | 0.00981269 | 15 |
| *lightgreen* | GO:0010257 | NADH dehydrogenase complex assembly | 0.01180231 | 6 |
| *lightgreen* | GO:0032981 | mitochondrial respiratory chain complex I assembly | 0.01180231 | 6 |
| *lightgreen* | GO:0019693 | ribose phosphate metabolic process | 0.01287405 | 15 |
| *lightgreen* | GO:0015985 | energy coupled proton transport, down electrochemical gradient | 0.01287405 | 4 |
| *lightgreen* | GO:0015986 | ATP synthesis coupled proton transport | 0.01287405 | 4 |
| *lightgreen* | GO:0072521 | purine-containing compound metabolic process | 0.01400888 | 15 |
| *lightgreen* | GO:0006415 | translational termination | 0.0198795 | 7 |
| *lightgreen* | GO:0017144 | drug metabolic process | 0.0198795 | 17 |
| *lightgreen* | GO:0006368 | transcription elongation from RNA polymerase II promoter | 0.02980306 | 6 |
| *lightgreen* | GO:1902600 | proton transmembrane transport | 0.03606153 | 7 |
| *lightgreen* | GO:0070125 | mitochondrial translational elongation | 0.04908994 | 6 |
| *midnightblue* | GO:0009226 | nucleotide-sugar biosynthetic process | 0.02485856 | 5 |
| *purple* | GO:0030036 | actin cytoskeleton organization | 0.00017442 | 28 |
| *purple* | GO:0043299 | leukocyte degranulation | 0.00017442 | 31 |
| *purple* | GO:0002275 | myeloid cell activation involved in immune response | 0.00017442 | 31 |
| *purple* | GO:0030029 | actin filament-based process | 0.00017442 | 29 |
| *purple* | GO:0036230 | granulocyte activation | 0.00017442 | 30 |
| *purple* | GO:0002444 | myeloid leukocyte mediated immunity | 0.00017442 | 31 |
| *purple* | GO:0002283 | neutrophil activation involved in immune response | 0.00022617 | 29 |
| *purple* | GO:0002446 | neutrophil mediated immunity | 0.00024568 | 29 |
| *purple* | GO:0042119 | neutrophil activation | 0.00024568 | 29 |
| *purple* | GO:0043312 | neutrophil degranulation | 0.00046569 | 28 |
| *purple* | GO:0051656 | establishment of organelle localization | 0.00297796 | 22 |
| *purple* | GO:0030834 | regulation of actin filament depolymerization | 0.00358862 | 7 |
| *purple* | GO:0051016 | barbed-end actin filament capping | 0.00473557 | 5 |
| *purple* | GO:0007015 | actin filament organization | 0.00473557 | 18 |
| *purple* | GO:0030042 | actin filament depolymerization | 0.00507899 | 7 |
| *purple* | GO:0032956 | regulation of actin cytoskeleton organization | 0.00507899 | 16 |
| *purple* | GO:0097435 | supramolecular fiber organization | 0.00507899 | 23 |
| *purple* | GO:0008154 | actin polymerization or depolymerization | 0.00573309 | 13 |
| *purple* | GO:0051640 | organelle localization | 0.00975306 | 26 |
| *purple* | GO:0015991 | ATP hydrolysis coupled proton transport | 0.01105061 | 5 |
| *purple* | GO:0032970 | regulation of actin filament-based process | 0.01130579 | 16 |
| *purple* | GO:0061615 | glycolytic process through fructose-6-phosphate | 0.01487117 | 5 |
| *purple* | GO:0061620 | glycolytic process through glucose-6-phosphate | 0.01487117 | 5 |
| *purple* | GO:0090662 | ATP hydrolysis coupled transmembrane transport | 0.01487117 | 5 |
| *purple* | GO:0099131 | ATP hydrolysis coupled ion transmembrane transport | 0.01487117 | 5 |
| *purple* | GO:1901879 | regulation of protein depolymerization | 0.01682422 | 7 |
| *purple* | GO:0008064 | regulation of actin polymerization or depolymerization | 0.01682422 | 11 |
| *purple* | GO:0030832 | regulation of actin filament length | 0.01682422 | 11 |
| *purple* | GO:0030041 | actin filament polymerization | 0.01749462 | 11 |
| *purple* | GO:0060627 | regulation of vesicle-mediated transport | 0.01846772 | 19 |
| *purple* | GO:0010591 | regulation of lamellipodium assembly | 0.01846772 | 5 |
| *purple* | GO:0015988 | energy coupled proton transmembrane transport, against electrochemical gradient | 0.01846772 | 5 |
| *purple* | GO:0002429 | immune response-activating cell surface receptor signaling pathway | 0.02205143 | 16 |
| *purple* | GO:0002260 | lymphocyte homeostasis | 0.02456769 | 7 |
| *purple* | GO:0051493 | regulation of cytoskeleton organization | 0.02550649 | 19 |
| *purple* | GO:0051693 | actin filament capping | 0.03024758 | 5 |
| *purple* | GO:0016236 | macroautophagy | 0.03257191 | 16 |
| *purple* | GO:0033572 | transferrin transport | 0.03448228 | 5 |
| *purple* | GO:0006897 | endocytosis | 0.03637403 | 23 |
| *purple* | GO:0015682 | ferric iron transport | 0.03637403 | 5 |
| *purple* | GO:0030835 | negative regulation of actin filament depolymerization | 0.03637403 | 5 |
| *purple* | GO:0071242 | cellular response to ammonium ion | 0.03637403 | 5 |
| *purple* | GO:0072512 | trivalent inorganic cation transport | 0.03637403 | 5 |
| *purple* | GO:0006909 | phagocytosis | 0.03990427 | 13 |
| *purple* | GO:0110053 | regulation of actin filament organization | 0.03990427 | 12 |
| *purple* | GO:0006734 | NADH metabolic process | 0.03990427 | 5 |
| *purple* | GO:0007266 | Rho protein signal transduction | 0.03990427 | 10 |
| *purple* | GO:0016241 | regulation of macroautophagy | 0.03990427 | 11 |
| *purple* | GO:0002768 | immune response-regulating cell surface receptor signaling pathway | 0.04245297 | 16 |
| *purple* | GO:0048771 | tissue remodeling | 0.04245297 | 8 |
| *purple* | GO:0051261 | protein depolymerization | 0.04744838 | 7 |
| *purple* | GO:0022604 | regulation of cell morphogenesis | 0.04744838 | 16 |
| *purple* | GO:1902743 | regulation of lamellipodium organization | 0.04809712 | 5 |
| *saddlebrown* | GO:0007596 | blood coagulation | 2.35E-15 | 20 |
| *saddlebrown* | GO:0050817 | coagulation | 2.35E-15 | 20 |
| *saddlebrown* | GO:0007599 | hemostasis | 2.35E-15 | 20 |
| *saddlebrown* | GO:0042060 | wound healing | 1.40E-14 | 22 |
| *saddlebrown* | GO:0009611 | response to wounding | 3.29E-14 | 23 |
| *saddlebrown* | GO:0030168 | platelet activation | 1.24E-13 | 15 |
| *saddlebrown* | GO:0050878 | regulation of body fluid levels | 2.35E-13 | 20 |
| *saddlebrown* | GO:0002576 | platelet degranulation | 3.56E-08 | 10 |
| *saddlebrown* | GO:0070527 | platelet aggregation | 2.20E-06 | 7 |
| *saddlebrown* | GO:0034109 | homotypic cell-cell adhesion | 6.05E-06 | 7 |
| *saddlebrown* | GO:0098609 | cell-cell adhesion | 9.89E-05 | 14 |
| *saddlebrown* | GO:0072376 | protein activation cascade | 0.0004064 | 5 |
| *saddlebrown* | GO:0061041 | regulation of wound healing | 0.00042858 | 6 |
| *saddlebrown* | GO:0030193 | regulation of blood coagulation | 0.00043564 | 5 |
| *saddlebrown* | GO:1900046 | regulation of hemostasis | 0.00043564 | 5 |
| *saddlebrown* | GO:0050818 | regulation of coagulation | 0.00046962 | 5 |
| *saddlebrown* | GO:1903034 | regulation of response to wounding | 0.00093943 | 6 |
| *saddlebrown* | GO:0019730 | antimicrobial humoral response | 0.00100051 | 5 |
| *saddlebrown* | GO:0061844 | antimicrobial humoral immune response mediated by antimicrobial peptide | 0.00248972 | 4 |
| *saddlebrown* | GO:0050900 | leukocyte migration | 0.00350938 | 9 |
| *saddlebrown* | GO:0030219 | megakaryocyte differentiation | 0.00411546 | 5 |
| *saddlebrown* | GO:0006936 | muscle contraction | 0.00411546 | 7 |
| *saddlebrown* | GO:0006937 | regulation of muscle contraction | 0.00530278 | 5 |
| *saddlebrown* | GO:0010543 | regulation of platelet activation | 0.01402396 | 3 |
| *saddlebrown* | GO:0030195 | negative regulation of blood coagulation | 0.01472732 | 3 |
| *saddlebrown* | GO:0050819 | negative regulation of coagulation | 0.01472732 | 3 |
| *saddlebrown* | GO:1900047 | negative regulation of hemostasis | 0.01472732 | 3 |
| *saddlebrown* | GO:0030198 | extracellular matrix organization | 0.01749332 | 6 |
| *saddlebrown* | GO:0045652 | regulation of megakaryocyte differentiation | 0.01749332 | 4 |
| *saddlebrown* | GO:0030593 | neutrophil chemotaxis | 0.01828214 | 4 |
| *saddlebrown* | GO:0003012 | muscle system process | 0.01843488 | 7 |
| *saddlebrown* | GO:0019932 | second-messenger-mediated signaling | 0.01843488 | 7 |
| *saddlebrown* | GO:0019369 | arachidonic acid metabolic process | 0.02157555 | 3 |
| *saddlebrown* | GO:0050891 | multicellular organismal water homeostasis | 0.02691885 | 3 |
| *saddlebrown* | GO:1990266 | neutrophil migration | 0.02951413 | 4 |
| *saddlebrown* | GO:0090303 | positive regulation of wound healing | 0.02951413 | 3 |
| *saddlebrown* | GO:0071621 | granulocyte chemotaxis | 0.02951413 | 4 |
| *saddlebrown* | GO:0043062 | extracellular structure organization | 0.02951413 | 6 |
| *saddlebrown* | GO:0007611 | learning or memory | 0.02951413 | 5 |
| *saddlebrown* | GO:0006959 | humoral immune response | 0.02951413 | 5 |
| *saddlebrown* | GO:0007612 | learning | 0.02951413 | 4 |
| *saddlebrown* | GO:0030104 | water homeostasis | 0.02951413 | 3 |
| *saddlebrown* | GO:0061045 | negative regulation of wound healing | 0.02951413 | 3 |
| *saddlebrown* | GO:0090257 | regulation of muscle system process | 0.02951413 | 5 |
| *saddlebrown* | GO:0007229 | integrin-mediated signaling pathway | 0.03500369 | 4 |
| *saddlebrown* | GO:1903036 | positive regulation of response to wounding | 0.03770901 | 3 |
| *saddlebrown* | GO:0007160 | cell-matrix adhesion | 0.04253159 | 5 |
| *saddlebrown* | GO:0070098 | chemokine-mediated signaling pathway | 0.04253159 | 3 |
| *saddlebrown* | GO:1903035 | negative regulation of response to wounding | 0.04253159 | 3 |
| *saddlebrown* | GO:0019935 | cyclic-nucleotide-mediated signaling | 0.0426938 | 4 |
| *saddlebrown* | GO:0031589 | cell-substrate adhesion | 0.0426938 | 6 |
| *saddlebrown* | GO:0097530 | granulocyte migration | 0.043736 | 4 |
| *saddlebrown* | GO:0050890 | cognition | 0.043736 | 5 |
| *skyblue* | GO:0006614 | SRP-dependent cotranslational protein targeting to membrane | 8.75E-25 | 21 |
| *skyblue* | GO:0006613 | cotranslational protein targeting to membrane | 8.75E-25 | 21 |
| *skyblue* | GO:0000184 | nuclear-transcribed mRNA catabolic process, nonsense-mediated decay | 8.75E-25 | 22 |
| *skyblue* | GO:0045047 | protein targeting to ER | 3.68E-24 | 21 |
| *skyblue* | GO:0072599 | establishment of protein localization to endoplasmic reticulum | 5.56E-24 | 21 |
| *skyblue* | GO:0006413 | translational initiation | 1.48E-22 | 23 |
| *skyblue* | GO:0070972 | protein localization to endoplasmic reticulum | 1.48E-22 | 21 |
| *skyblue* | GO:0006612 | protein targeting to membrane | 2.65E-20 | 21 |
| *skyblue* | GO:0000956 | nuclear-transcribed mRNA catabolic process | 4.35E-20 | 22 |
| *skyblue* | GO:0006401 | RNA catabolic process | 3.77E-17 | 23 |
| *skyblue* | GO:0006402 | mRNA catabolic process | 8.71E-17 | 22 |
| *skyblue* | GO:0090150 | establishment of protein localization to membrane | 2.40E-16 | 21 |
| *skyblue* | GO:0006605 | protein targeting | 4.60E-16 | 23 |
| *skyblue* | GO:1901361 | organic cyclic compound catabolic process | 7.41E-14 | 24 |
| *skyblue* | GO:0072594 | establishment of protein localization to organelle | 7.66E-14 | 23 |
| *skyblue* | GO:0034655 | nucleobase-containing compound catabolic process | 1.36E-13 | 23 |
| *skyblue* | GO:0046700 | heterocycle catabolic process | 3.23E-13 | 23 |
| *skyblue* | GO:0044270 | cellular nitrogen compound catabolic process | 3.23E-13 | 23 |
| *skyblue* | GO:0019439 | aromatic compound catabolic process | 4.01E-13 | 23 |
| *skyblue* | GO:0072657 | protein localization to membrane | 4.30E-12 | 21 |
| *skyblue* | GO:0022613 | ribonucleoprotein complex biogenesis | 9.07E-06 | 14 |
| *skyblue* | GO:0042254 | ribosome biogenesis | 2.96E-05 | 11 |
| *skyblue* | GO:0022618 | ribonucleoprotein complex assembly | 3.22E-05 | 10 |
| *skyblue* | GO:0002181 | cytoplasmic translation | 3.22E-05 | 7 |
| *skyblue* | GO:0000027 | ribosomal large subunit assembly | 3.50E-05 | 5 |
| *skyblue* | GO:0071826 | ribonucleoprotein complex subunit organization | 4.94E-05 | 10 |
| *skyblue* | GO:0042255 | ribosome assembly | 5.59E-05 | 6 |
| *skyblue* | GO:0042273 | ribosomal large subunit biogenesis | 0.00013099 | 6 |
| *skyblue* | GO:0001937 | negative regulation of endothelial cell proliferation | 0.00691503 | 3 |
| *skyblue* | GO:0006364 | rRNA processing | 0.00759955 | 7 |
| *skyblue* | GO:0016072 | rRNA metabolic process | 0.01779429 | 7 |
| *skyblue* | GO:0042274 | ribosomal small subunit biogenesis | 0.01779429 | 4 |
| *skyblue* | GO:0000462 | maturation of SSU-rRNA from tricistronic rRNA transcript (SSU-rRNA, 5.8S rRNA, LSU-rRNA) | 0.03461278 | 3 |
| *skyblue3* | GO:0060260 | regulation of transcription initiation from RNA polymerase II promoter | 0.0410107 | 3 |
| *skyblue3* | GO:0042026 | protein refolding | 0.0410107 | 3 |
| *skyblue3* | GO:2000142 | regulation of DNA-templated transcription, initiation | 0.0410107 | 3 |

**Table S7. Unique genes, *Candida* vs other pathogen classes**

| **GENEID** | **SYMBOL** |
| --- | --- |
| ENSG00000241294 | *IGKV2-24* |
| ENSG00000224650 | *IGHV3-74* |
| ENSG00000117399 | *CDC20* |
| ENSG00000148773 | *MKI67* |
| ENSG00000111206 | *FOXM1* |
| ENSG00000089685 | *BIRC5* |
| ENSG00000088325 | *TPX2* |
| ENSG00000145423 | *SFRP2* |
| ENSG00000167900 | *TK1* |
| ENSG00000237649 | *KIFC1* |
| ENSG00000171848 | *RRM2* |
| ENSG00000211649 | *IGLV7-46* |
| ENSG00000176890 | *TYMS* |
| ENSG00000127564 | *PKMYT1* |
| ENSG00000169385 | *RNASE2* |
| ENSG00000198959 | *TGM2* |
| ENSG00000101057 | *MYBL2* |
| ENSG00000092067 | *CEBPE* |
| ENSG00000235169 | *SMIM1* |
| ENSG00000164611 | *PTTG1* |
| ENSG00000178999 | *AURKB* |
| ENSG00000278196 | *IGLV2-8* |
| ENSG00000076382 | *SPAG5* |
| ENSG00000211938 | *IGHV3-7* |
| ENSG00000172889 | *EGFL7* |
| ENSG00000197582 | *GPX1P1* |
| ENSG00000162366 | *PDZK1IP1* |
| ENSG00000136732 | *GYPC* |
| ENSG00000011028 | *MRC2* |
| ENSG00000088992 | *TESC* |
| ENSG00000099194 | *SCD* |
| ENSG00000166851 | *PLK1* |
| ENSG00000182732 | *RGS6* |
| ENSG00000196465 | *MYL6B* |
| ENSG00000115641 | *FHL2* |
| ENSG00000166091 | *CMTM5* |
| ENSG00000211962 | *IGHV1-46* |
| ENSG00000185499 | *MUC1* |
| ENSG00000175063 | *UBE2C* |
| ENSG00000143774 | *GUK1* |
| ENSG00000168528 | *SERINC2* |
| ENSG00000240583 | *AQP1* |
| ENSG00000161911 | *TREML1* |
| ENSG00000205309 | *NT5M* |
| ENSG00000104903 | *LYL1* |
| ENSG00000212864 | *RNF208* |
| ENSG00000101412 | *E2F1* |
| ENSG00000159335 | *PTMS* |
| ENSG00000146918 | *NCAPG2* |
| ENSG00000246705 | *H2AFJ* |
| ENSG00000181218 | *HIST3H2A* |
| ENSG00000125037 | *EMC3* |
| ENSG00000070404 | *FSTL3* |
| ENSG00000170522 | *ELOVL6* |
| ENSG00000205726 | *ITSN1* |
| ENSG00000126088 | *UROD* |
| ENSG00000100116 | *GCAT* |
| ENSG00000161944 | *ASGR2* |
| ENSG00000106462 | *EZH2* |
| ENSG00000131462 | *TUBG1* |
| ENSG00000179862 | *CITED4* |
| ENSG00000117592 | *PRDX6* |
| ENSG00000099785 | *MARCH2* |
| ENSG00000117984 | *CTSD* |
| ENSG00000126267 | *COX6B1* |
| ENSG00000125995 | *ROMO1* |
| ENSG00000072954 | *TMEM38A* |
| ENSG00000103876 | *FAH* |
| ENSG00000082781 | *ITGB5* |
| ENSG00000177697 | *CD151* |
| ENSG00000122490 | *PQLC1* |
| ENSG00000067836 | *ROGDI* |
| ENSG00000100979 | *PLTP* |
| ENSG00000076003 | *MCM6* |
| ENSG00000142669 | *SH3BGRL3* |
| ENSG00000185340 | *GAS2L1* |
| ENSG00000237181 | *AC147651.4* |
| ENSG00000074416 | *MGLL* |
| ENSG00000075188 | *NUP37* |
| ENSG00000165914 | *TTC7B* |
| ENSG00000003147 | *ICA1* |
| ENSG00000142733 | *MAP3K6* |
| ENSG00000280543 | *ASAP1-IT2* |
| ENSG00000197858 | *GPAA1* |
| ENSG00000087086 | *FTL* |
| ENSG00000064490 | *RFXANK* |
| ENSG00000102265 | *TIMP1* |
| ENSG00000110011 | *DNAJC4* |
| ENSG00000148180 | *GSN* |
| ENSG00000183963 | *SMTN* |
| ENSG00000175416 | *CLTB* |
| ENSG00000136930 | *PSMB7* |
| ENSG00000142507 | *PSMB6* |
| ENSG00000196154 | *S100A4* |
| ENSG00000131495 | *NDUFA2* |
| ENSG00000160211 | *G6PD* |
| ENSG00000133246 | *PRAM1* |
| ENSG00000204463 | *BAG6* |
| ENSG00000164978 | *NUDT2* |
| ENSG00000180185 | *FAHD1* |
| ENSG00000175294 | *CATSPER1* |
| ENSG00000143537 | *ADAM15* |
| ENSG00000116691 | *MIIP* |
| ENSG00000128309 | *MPST* |
| ENSG00000177700 | *POLR2L* |
| ENSG00000126005 | *MMP24-AS1* |
| ENSG00000172428 | *COPS9* |
| ENSG00000162129 | *CLPB* |
| ENSG00000141552 | *ANAPC11* |
| ENSG00000106400 | *ZNHIT1* |
| ENSG00000170043 | *TRAPPC1* |
| ENSG00000105669 | *COPE* |
| ENSG00000115350 | *POLE4* |
| ENSG00000170310 | *STX8* |
| ENSG00000102032 | *RENBP* |
| ENSG00000126934 | *MAP2K2* |
| ENSG00000126768 | *TIMM17B* |
| ENSG00000185624 | *P4HB* |
| ENSG00000254999 | *BRK1* |
| ENSG00000197982 | *C1orf122* |
| ENSG00000168894 | *RNF181* |
| ENSG00000169976 | *SF3B5* |
| ENSG00000140553 | *UNC45A* |
| ENSG00000116586 | *LAMTOR2* |
| ENSG00000167553 | *TUBA1C* |
| ENSG00000104774 | *MAN2B1* |
| ENSG00000162073 | *PAQR4* |
| ENSG00000108518 | *PFN1* |
| ENSG00000204428 | *LY6G5C* |
| ENSG00000127838 | *PNKD* |
| ENSG00000135940 | *COX5B* |
| ENSG00000171984 | *C20orf196* |
| ENSG00000187051 | *RPS19BP1* |
| ENSG00000137076 | *TLN1* |
| ENSG00000173264 | *GPR137* |
| ENSG00000163374 | *YY1AP1* |
| ENSG00000169919 | *GUSB* |
| ENSG00000269743 | *SLC25A53* |
| ENSG00000171298 | *GAA* |
| ENSG00000002330 | *BAD* |
| ENSG00000175221 | *MED16* |
| ENSG00000167264 | *DUS2* |
| ENSG00000005075 | *POLR2J* |
| ENSG00000176783 | *RUFY1* |
| ENSG00000077150 | *NFKB2* |
| ENSG00000149761 | *NUDT22* |
| ENSG00000111775 | *COX6A1* |
| ENSG00000174891 | *RSRC1* |
| ENSG00000204498 | *NFKBIL1* |
| ENSG00000126522 | *ASL* |
| ENSG00000109685 | *WHSC1* |
| ENSG00000213523 | *SRA1* |
| ENSG00000168818 | *STX18* |
| ENSG00000163479 | *SSR2* |
| ENSG00000172590 | *MRPL52* |
| ENSG00000105723 | *GSK3A* |
| ENSG00000106367 | *AP1S1* |
| ENSG00000162236 | *STX5* |
| ENSG00000129219 | *PLD2* |
| ENSG00000079459 | *FDFT1* |
| ENSG00000172586 | *CHCHD1* |
| ENSG00000127774 | *EMC6* |
| ENSG00000008018 | *PSMB1* |
| ENSG00000135506 | *OS9* |
| ENSG00000105254 | *TBCB* |
| ENSG00000166337 | *TAF10* |
| ENSG00000141759 | *TXNL4A* |
| ENSG00000134030 | *CTIF* |
| ENSG00000084693 | *AGBL5* |
| ENSG00000084754 | *HADHA* |
| ENSG00000214078 | *CPNE1* |
| ENSG00000130517 | *PGPEP1* |
| ENSG00000140374 | *ETFA* |
| ENSG00000161013 | *MGAT4B* |
| ENSG00000123144 | *C19orf43* |
| ENSG00000159210 | *SNF8* |
| ENSG00000167863 | *ATP5H* |
| ENSG00000102879 | *CORO1A* |
| ENSG00000143420 | *ENSA* |
| ENSG00000167674 | *CTB-50L17.10* |
| ENSG00000130985 | *UBA1* |
| ENSG00000175826 | *CTDNEP1* |
| ENSG00000109111 | *SUPT6H* |
| ENSG00000105401 | *CDC37* |
| ENSG00000101294 | *HM13* |
| ENSG00000078668 | *VDAC3* |
| ENSG00000130402 | *ACTN4* |
| ENSG00000185627 | *PSMD13* |
| ENSG00000125912 | *NCLN* |
| ENSG00000165280 | *VCP* |
| ENSG00000180104 | *EXOC3* |
| ENSG00000182180 | *MRPS16* |
| ENSG00000126062 | *TMEM115* |
| ENSG00000170144 | *HNRNPA3* |
| ENSG00000086589 | *RBM22* |
| ENSG00000163161 | *ERCC3* |
| ENSG00000125447 | *GGA3* |
| ENSG00000117748 | *RPA2* |
| ENSG00000084090 | *STARD7* |
| ENSG00000102572 | *STK24* |
| ENSG00000119772 | *DNMT3A* |
| ENSG00000068745 | *IP6K2* |
| ENSG00000204599 | *TRIM39* |
| ENSG00000112855 | *HARS2* |
| ENSG00000163219 | *ARHGAP25* |
| ENSG00000102978 | *POLR2C* |
| ENSG00000204261 | *PSMB8-AS1* |
| ENSG00000121774 | *KHDRBS1* |
| ENSG00000247315 | *ZCCHC3* |
| ENSG00000166170 | *BAG5* |
| ENSG00000147789 | *ZNF7* |
| ENSG00000117751 | *PPP1R8* |
| ENSG00000107263 | *RAPGEF1* |
| ENSG00000153560 | *UBP1* |
| ENSG00000123595 | *RAB9A* |
| ENSG00000197857 | *ZNF44* |
| ENSG00000198728 | *LDB1* |
| ENSG00000140577 | *CRTC3* |
| ENSG00000196182 | *STK40* |
| ENSG00000158467 | *AHCYL2* |
| ENSG00000183309 | *ZNF623* |
| ENSG00000119523 | *ALG2* |
| ENSG00000146833 | *TRIM4* |
| ENSG00000034533 | *ASTE1* |
| ENSG00000083844 | *ZNF264* |
| ENSG00000183864 | *TOB2* |
| ENSG00000140943 | *MBTPS1* |
| ENSG00000155393 | *HEATR3* |
| ENSG00000064703 | *DDX20* |
| ENSG00000241878 | *PISD* |
| ENSG00000140987 | *ZSCAN32* |
| ENSG00000114503 | *NCBP2* |
| ENSG00000168906 | *MAT2A* |
| ENSG00000142528 | *ZNF473* |
| ENSG00000061794 | *MRPS35* |
| ENSG00000008869 | *HEATR5B* |
| ENSG00000105879 | *CBLL1* |
| ENSG00000256683 | *ZNF350* |
| ENSG00000150401 | *DCUN1D2* |
| ENSG00000096746 | *HNRNPH3* |
| ENSG00000181896 | *ZNF101* |
| ENSG00000131115 | *ZNF227* |
| ENSG00000143149 | *ALDH9A1* |
| ENSG00000134283 | *PPHLN1* |
| ENSG00000197183 | *NOL4L* |
| ENSG00000197037 | *ZSCAN25* |
| ENSG00000151576 | *QTRT2* |
| ENSG00000167987 | *VPS37C* |
| ENSG00000171469 | *ZNF561* |
| ENSG00000198783 | *ZNF830* |
| ENSG00000148153 | *INIP* |
| ENSG00000033030 | *ZCCHC8* |
| ENSG00000119537 | *KDSR* |
| ENSG00000163684 | *RPP14* |
| ENSG00000124177 | *CHD6* |
| ENSG00000165943 | *MOAP1* |
| ENSG00000083814 | *ZNF671* |
| ENSG00000139620 | *KANSL2* |
| ENSG00000253719 | *ATXN7L3B* |
| ENSG00000163545 | *NUAK2* |
| ENSG00000163608 | *NEPRO* |
| ENSG00000162976 | *PQLC3* |
| ENSG00000172175 | *MALT1* |
| ENSG00000164091 | *WDR82* |
| ENSG00000011523 | *CEP68* |
| ENSG00000107290 | *SETX* |
| ENSG00000170949 | *ZNF160* |
| ENSG00000181472 | *ZBTB2* |
| ENSG00000240038 | *AMY2B* |
| ENSG00000149716 | *ORAOV1* |
| ENSG00000180228 | *PRKRA* |
| ENSG00000136720 | *HS6ST1* |
| ENSG00000124217 | *MOCS3* |
| ENSG00000198482 | *ZNF808* |
| ENSG00000163328 | *GPR155* |
| ENSG00000139197 | *PEX5* |
| ENSG00000132680 | *KIAA0907* |
| ENSG00000151466 | *SCLT1* |
| ENSG00000205268 | *PDE7A* |
| ENSG00000171823 | *FBXL14* |
| ENSG00000281026 | *N4BP2L2-IT2* |
| ENSG00000263753 | *LINC00667* |
| ENSG00000077713 | *SLC25A43* |
| ENSG00000112763 | *BTN2A1* |
| ENSG00000167635 | *ZNF146* |
| ENSG00000167232 | *ZNF91* |
| ENSG00000150995 | *ITPR1* |
| ENSG00000224660 | *SH3BP5-AS1* |
| ENSG00000180787 | *ZFP3* |
| ENSG00000132952 | *USPL1* |
| ENSG00000165359 | *INTS6L* |
| ENSG00000135378 | *PRRG4* |
| ENSG00000147679 | *UTP23* |
| ENSG00000073417 | *PDE8A* |
| ENSG00000197608 | *ZNF841* |
| ENSG00000089022 | *MAPKAPK5* |
| ENSG00000169598 | *DFFB* |
| ENSG00000158158 | *CNNM4* |
| ENSG00000175787 | *ZNF169* |
| ENSG00000196670 | *ZFP62* |
| ENSG00000101665 | *SMAD7* |
| ENSG00000172243 | *CLEC7A* |
| ENSG00000164144 | *ARFIP1* |
| ENSG00000159388 | *BTG2* |
| ENSG00000109689 | *STIM2* |
| ENSG00000107957 | *SH3PXD2A* |
| ENSG00000174796 | *THAP6* |
| ENSG00000184588 | *PDE4B* |
| ENSG00000181982 | *CCDC149* |
| ENSG00000285589 | *AC010422.8* |
| ENSG00000223547 | *ZNF844* |
| ENSG00000188785 | *ZNF548* |
| ENSG00000181894 | *ZNF329* |
| ENSG00000196387 | *ZNF140* |
| ENSG00000182782 | *HCAR2* |
| ENSG00000101596 | *SMCHD1* |
| ENSG00000155621 | *C9orf85* |
| ENSG00000235859 | *AC006978.6* |
| ENSG00000196646 | *ZNF136* |
| ENSG00000139318 | *DUSP6* |
| ENSG00000269713 | *NBPF9* |
| ENSG00000171643 | *S100Z* |
| ENSG00000261087 | *KB-1460A1.5* |
| ENSG00000105708 | *ZNF14* |
| ENSG00000162885 | *B3GALNT2* |
| ENSG00000257027 | *RP11-705C15.3* |
| ENSG00000008256 | *CYTH3* |
| ENSG00000154059 | *IMPACT* |
| ENSG00000234444 | *ZNF736* |
| ENSG00000237440 | *ZNF737* |
| ENSG00000167981 | *ZNF597* |
| ENSG00000274021 | *RP11-823E8.3* |
| ENSG00000109618 | *SEPSECS* |
| ENSG00000120458 | *MSANTD2* |
| ENSG00000073464 | *CLCN4* |
| ENSG00000204130 | *RUFY2* |
| ENSG00000196470 | *SIAH1* |
| ENSG00000156738 | *MS4A1* |
| ENSG00000164180 | *TMEM161B* |
| ENSG00000113369 | *ARRDC3* |
| ENSG00000167384 | *ZNF180* |
| ENSG00000144130 | *NT5DC4* |

**Table S8. Top 5 functional annotation clusters, *Candida* vs other pathogen classes**

| Functional Annotation Clustering | P value |
| --- | --- |
| ***Cluster 1 – Enrichment Score 4.85*** |  |
| **Antibacterial humoral response**  **Innate immune response in mucosa**  **Defense response to gram-positive bacterium** | 8.4E-5  2.1E-3  1.1E-2 |
| ***Cluster 2 – Enrichment Score 4.34*** |  |
| **Hemoglobin’s chaperone**  **Hemoglobin complex**  **Iron ion binding**  **Haptoglobin-hemoglobin complex**  **Oxygen transporter activity**  **Hydrogen peroxide catabolic process**  **Positive regulation of cell death** | 7.1E-10  7.2E-8  3.4E-6  4.0E-6  9.2E-6  4.5E-5  3.2E-3 |
| ***Cluster 3 – Enrichment Score 2.99*** |  |
| **Extracellular space**  **Signal peptide** | 1.7E-7  3.1E-3 |
| ***Cluster 4 – Enrichment Score 2.66*** |  |
| **Negative regulation of growth of symbiont in host**  **Defense response to bacterium**  **Response to yeast** | 5.4E-4  3.8E-3  7.6E-3 |
| ***Cluster 5 – Enrichment Score 2.31*** |  |
| **Heme biosynthetic process** | 2.5E-4 |

**Table S9. Confusion matrices for the multinomial classifier, demonstrating high test accuracy for all studied infection types and healthy controls. Rows represent true clinical phenotypes, columns represent predicted clinical phenotypes. A. Discovery cohort. B. Validation cohort.**

**A.**

|  |  | **Predicted** | | | | |  |
| --- | --- | --- | --- | --- | --- | --- | --- |
|  |  | **Bacterial** | **Candidemia** | **Healthy** | **SIRS** | **Viral** | **Percent Correct** |
| **True** | **Bacterial** | **31** | 4 | 0 | 0 | 0 | 89% |
|  | **Candidemia** | 1 | **19** | 0 | 2 | 1 | 83% |
|  | **Healthy** | 0 | 0 | **14** | 0 | 1 | 93% |
|  | **SIRS** | 0 | 1 | 0 | **15** | 1 | 88% |
|  | **Viral** | 0 | 0 | 0 | 1 | **47** | 98% |

**B.**

|  |  | **Predicted** | | | |  |
| --- | --- | --- | --- | --- | --- | --- |
|  |  | **Bacterial** | **Candidemia** | **Healthy** | **Viral** | **Percent Correct** |
| **True** | **Bacterial** | **8** | 0 | 0 | 1 | 89% |
|  | **Candidemia** | 0 | **18** | 0 | 1 | 95% |
|  | **Healthy** | 0 | 3 | **12** | 0 | 80% |
|  | **Viral** | 0 | 0 | 0 | **10** | 100% |

**Table S10. Signature performance is not associated with important clinical variables**

| **Variable** | **Correlation (ρ)** | **P value** |
| --- | --- | --- |
| Solid Organ Transplant | -0.2235 | 0.1548 |
| Cancer | -0.0022 | 0.9888 |
| QSOFA Score | 0.0710 | 0.6718 |
| White Blood Cell Count | -0.1767 | 0.2569 |
| Beta-d-glucan | 0.138 | 0.420 |

**Table S11. Ramilo, *et al.* Validation**

|  | |  |  |
| --- | --- | --- | --- |
|  |  | **Predicted** | |
|  |  | **Bacterial** | **Viral** |
| **True** | **Bacterial** | **71** | 1 |
|  | **Viral** | 3 | **15** |

**Table S12. Tsalik, *et al.* Validation**

|  | | **Predicted** | | |
| --- | --- | --- | --- | --- |
|  |  | **Bacterial** | **Viral** | **Noninfectious Illness** |
| **True** | **Bacterial** | **24** | 10 | 5 |
|  | **Viral** | 4 | **67** | 11 |
|  | **Noninfectious illness** | 4 | 11 | **57** |

**Table S13. PBMC Validation**

|  | | **Predicted** | | | |
| --- | --- | --- | --- | --- | --- |
|  |  | **Fungal** | **Bacterial** | **Viral** | **Uninfected** |
| **True** | **Fungal** | **16** | 1 | 0 | 1 |
|  | **Bacterial** | 1 | **16** | 0 | 1 |
|  | **Viral** | 0 | 0 | **5** | 1 |
|  | **Uninfected** | 2 | 2 | 0 | **2** |
