# Supplementary material for: The host transcriptional response to Candidemia is dominated by neutrophil activation and heme biosynthesis and supports novel diagnostic approaches
Source: Genome Med. 2021 Jul 5;13:108. doi: 10.1186/s13073-021-00924-9 (PMC8259367; doi:10.1186/s13073-021-00924-9)
Supplement: Supplementary file 2 — Additional file 2: Supplementary Figures S1-S4. Figure S1. Boxplot of the candidemia model predictive probabilities in the discovery and validation cohort by anti-fungal treatment. Figure S2. Differentially expressed genes in response to different infection phenotypes. Figure S3. Predictive Probability of Candidemia by Candida species. Figure S4. Signature performance with demographics included in the model. [file 13073_2021_924_MOESM2_ESM.docx]

**Supplementary Figures**

**
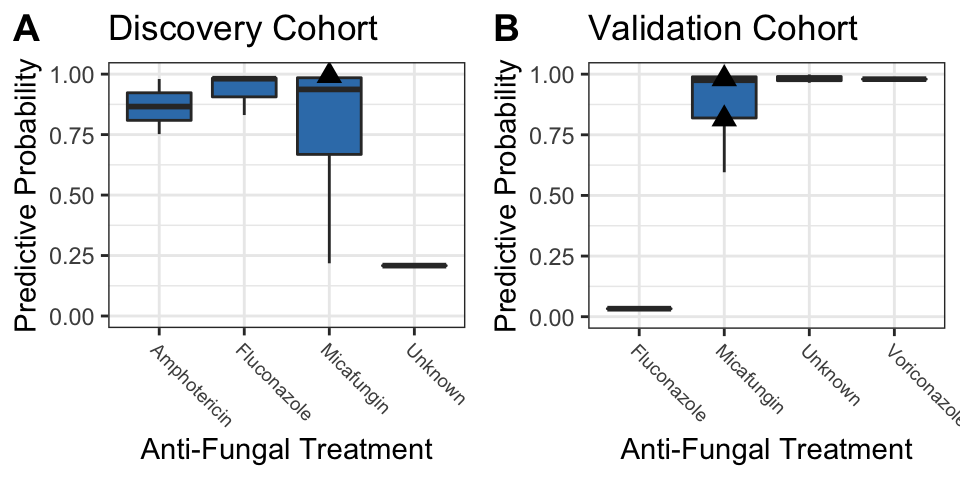
**

**Figure S1. Boxplot of the candidemia model predicted probabilities in the discovery (A) and validation (B) cohorts by anti-fungal treatment.** The triangles denote subjects that started treatment the day of sample collection.

**
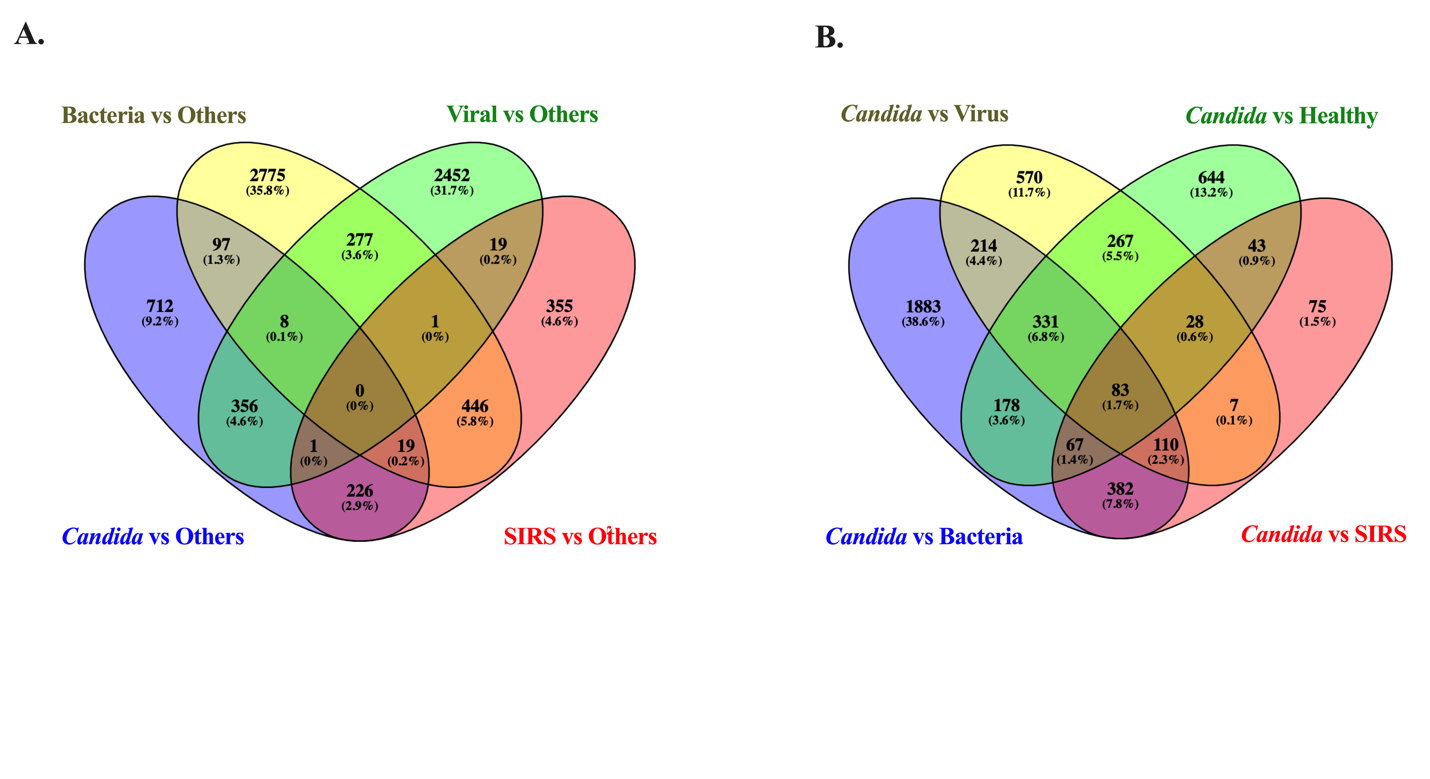
**

**Figure S2. Differentially expressed genes (adj P <0.05) in response to different infection phenotypes.** A. Only upregulated genes, infection phenotypes compared to all others. B. Only upregulated genes, *Candida* compared to each phenotype.

**
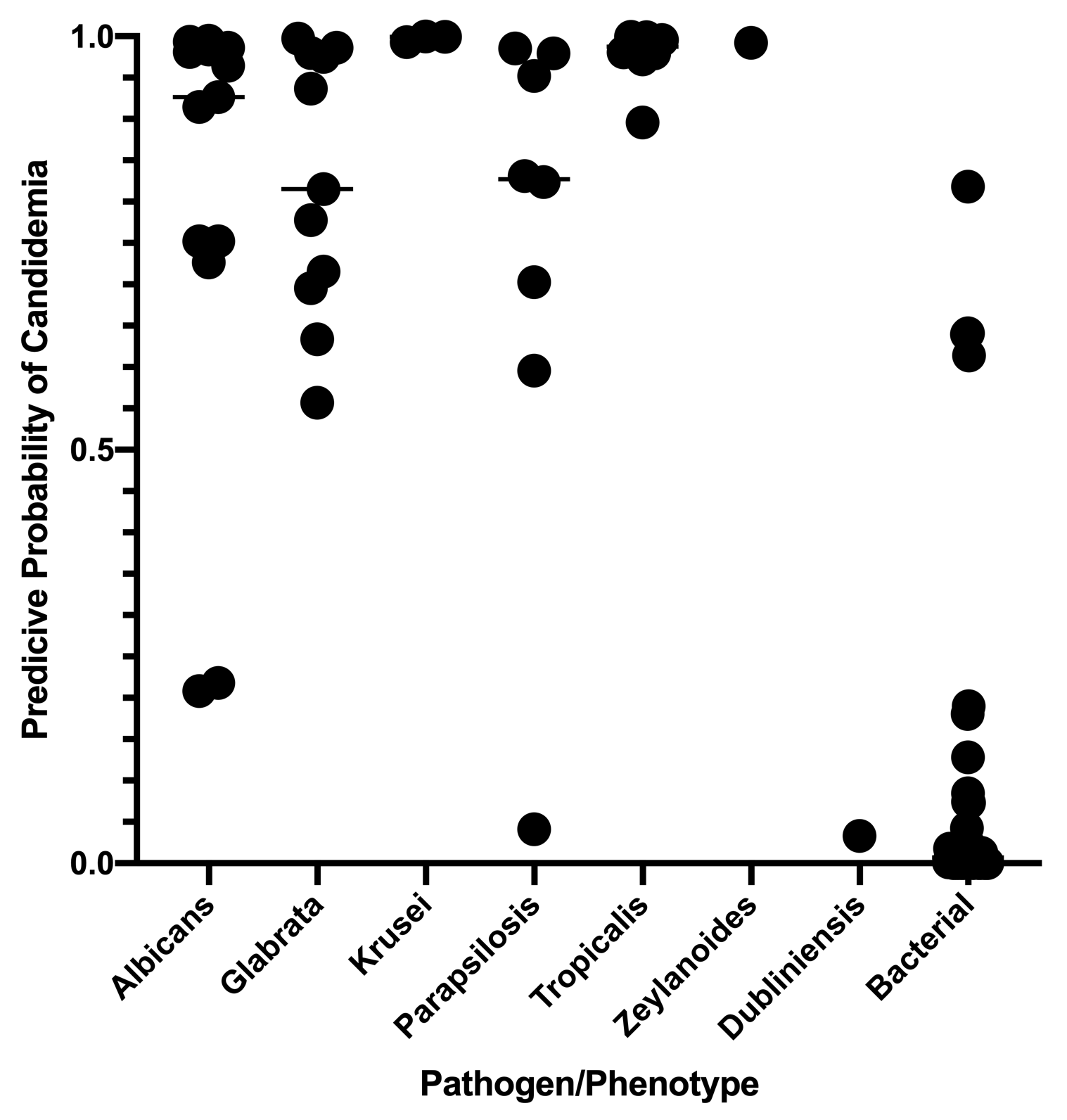
**

**Figure S3. Predictive Probability of Candidemia by *Candida* Species.** We examined the relationship between signature-derived predictive probability of candidemia and *Candida* species and found there was a statistically significant difference between group means as determined by one-way ANOVA (p=0.0234). On individual comparison between species, we found that only *C. tropicalis* demonstrated a statistically significant difference from the other *Candida* species (p=0.0446 *tropicalis* vs *albicans*, p=0.0203 *tropicalis* vs *glabrata,* p=0.007 *tropicalis* vs *parapsilosis*), although these differences did not affect overall accuracy of the signature across species. The data for the Bacterial phenotype is provided for comparison.


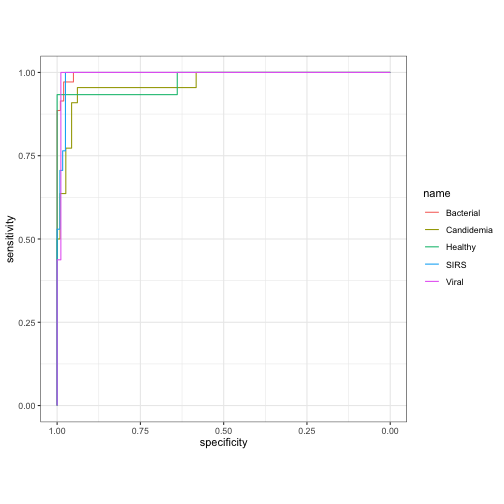


**Figure S4**. **Signature performance with demographics included in the model.** Compared to the original discovery signature, per-class rocAUCs are nearly identical (0.97 candidemia, 0.99 bacterial, 0.99 viral, 0.99 SIRS, 0.98 healthy).
